# Early Survival Prediction Framework in CD19-Specific CAR-T Cell Immunotherapy Using a Quantitative Systems Pharmacology Model

**DOI:** 10.3390/cancers13112782

**Published:** 2021-06-03

**Authors:** Anna Mueller-Schoell, Nahum Puebla-Osorio, Robin Michelet, Michael R. Green, Annette Künkele, Wilhelm Huisinga, Paolo Strati, Beth Chasen, Sattva S. Neelapu, Cassian Yee, Charlotte Kloft

**Affiliations:** 1 Department of Clinical Pharmacy and Biochemistry, Institute of Pharmacy, Freie Universitaet Berlin, 12169 Berlin, Germany; anna.mueller-schoell@fu-berlin.de (A.M.-S.); robin.michelet@fu-berlin.de (R.M.); 2 Graduate Research Training Program PharMetrX, 12169 Berlin, Germany; 3 Department of Lymphoma and Myeloma, Division of Cancer Medicine, The University of Texas MD Anderson Cancer Center, Houston, TX 77030, USA; npuebla@mdanderson.org (N.P.-O.); mgreen5@mdanderson.org (M.R.G.); PStrati@mdanderson.org (P.S.); 4 Department of Pediatric Oncology and Hematology, Charité–Universitätsmedizin Berlin, Corporate Member of Freie Universität Berlin, Humboldt–Universität zu Berlin, Augustenburger Platz 1, 1335 Berlin, Germany; annette.kuenkele@charite.de; 5 German Cancer Consortium (DKTK), Partner Site Berlin, CCC (Campus Mitte), 10178 Berlin, Germany; 6 Institute of Mathematics, University of Potsdam, 14476 Potsdam, Germany; huisinga@uni-potsdam.de; 7 Department of Nuclear Medicine, Division of Diagnostic Imaging, The University of Texas MD Anderson Cancer Center, Houston, TX 77030, USA; Beth.Chasen@mdanderson.org; 8 Department of Melanoma Medical Oncology, UT MD Anderson Cancer Center, Houston, TX 77030, USA; 9 Department of Immunology, UT MD Anderson Cancer Center, Houston, TX 70030, USA

**Keywords:** chimeric antigen receptor T cells, non-Hodgkin lymphoma, CAR-T cells, mathematical modeling, pharmacometrics

## Abstract

**Simple Summary:**

Treatment with chimeric antigen receptor (CAR)-T cells has improved the prognosis of patients with non-Hodgkin lymphoma (NHL) substantially. Yet, as up to 60% of patients eventually relapse, insights into factors determining treatment response are highly warranted. We used mathematical modeling to characterize typical and individual concentration–time profiles of four different CAR-T cell subtypes and tumor burden in 19 NHL patients and investigated patient-/therapy-related factors associated with poor survival. A low CAR-T cell maximum expansion capacity and no previous autologous stem cell transplantation were associated with a poor prognosis. We next translated our most important model parameter into a clinical composite score, which leverages but does not require the use of the model. Based on our clinical data, we propose a clinical composite score cut-off value for early survival prediction. Additional data will be needed to update and refine the developed model and the proposed clinical composite score cut-off value.

**Abstract:**

Chimeric antigen receptor (CAR)-T cell therapy has revolutionized treatment of relapsed/refractory non-Hodgkin lymphoma (NHL). However, since 36–60% of patients relapse, early response prediction is crucial. We present a novel population quantitative systems pharmacology model, integrating literature knowledge on physiology, immunology, and adoptive cell therapy together with 133 CAR-T cell phenotype, 1943 cytokine, and 48 metabolic tumor measurements. The model well described post-infusion concentrations of four CAR-T cell phenotypes and CD19^+^ metabolic tumor volume over 3 months after CAR-T cell infusion. Leveraging the model, we identified a low expansion subpopulation with significantly lower CAR-T cell expansion capacities amongst 19 NHL patients. Together with two patient-/therapy-related factors (autologous stem cell transplantation, CD4^+^/CD8^+^ T cells), the low expansion subpopulation explained 2/3 of the interindividual variability in the CAR-T cell expansion capacities. Moreover, the low expansion subpopulation had poor prognosis as only 1/4 of the low expansion subpopulation compared to 2/3 of the reference population were still alive after 24 months. We translated the expansion capacities into a clinical composite score (CCS) of ‘Maximum naïve CAR-T cell concentrations/Baseline tumor burden’ ratio and propose a CCS_TN_-value > 0.00136 (cells·µL^−1^·mL^−1^ as predictor for survival. Once validated in a larger cohort, the model will foster refining survival prediction and solutions to enhance NHL CAR-T cell therapy response.

## 1. Introduction

Chimeric antigen receptor (CAR)-T cell immunotherapy for relapsed or refractory B cell malignancies, such as acute lymphoblastic leukemia or non-Hodgkin lymphoma (NHL), has shown remarkable success [1,2,3,4]. Yet, in NHL, only 40–64% of patients show a durable response [1,5,6]. Thus, understanding the underlying factors of who will reach long-term response or not is essential. Several factors influence both CAR-T cell expansion and persistence, which are the two significant determinants of treatment response [7]. These factors include the design of the CAR construct [8], the manufacturing protocol [9], the frequency of functionally active T cells in the manufacturing and infusion products [10], the T cell phenotype [11,12], and the CD4^+^/CD8^+^ subset composition [12,13] in the infusion product, the tumor burden [10], the tumor microenvironment [14,15] and the dose and type of lymphodepleting chemotherapy [16,17]. Reported positive predictors for a long-term response are a high CAR-T cell maximum concentration (C_max_) [7,18,19], and a high area under the concentration–time curve in the first 28 days (AUC_0–28d_) [7]. Factors such as a high fraction of central memory T cells (T_CM_) or T memory stem cells (T_SCM_) in manufacturing and infusion product have been linked to a high expansion and persistence [11,20]. Yet there is still sizeable unexplained variability in expansion, persistence, and response amongst patients, i.e., interindividual variability [7].

Previous top-down and bottom-up mathematical models of CAR-T cell therapy have described the kinetics of CAR-T cells [21], the quantitative relationship between CAR-affinity, antigen abundance, tumor cell depletion, and CAR-T cell expansion [22], and the kinetics and dynamics of effector and memory CAR-T cells and tumor cells [23,24]. While ‘top-down’ models refer to models developed based on observed new data, ‘bottom-up’ models are human physiology- and drug characteristics-based models [25]. A ‘top-down’ approach might describe the observed CAR-T cell kinetic data well, yet, little information is gained about the underlying mechanisms, and prediction of a concentration–time profile for a new individual might not be informative. Bottom-up approaches can add in-depth insight into CAR-T cell-tumor dynamics on a cellular level but might be too complex for clinical use and often underestimate the interindividual variability. To explain parts of this variability and identify mechanistically plausible predictors for long-term response, we developed a population quantitative systems pharmacology (QSP) model, characterizing the human kinetics of four CAR-T cell phenotypes and CD19^+^ metabolic tumor volume and the dynamics of their interactions. Combining top-down and bottom-up approaches, our population QSP model uses prior information on CAR-T cell physiology together with in vivo data to inform unknown model parameter values and their interindividual variability [26]. Leveraging the model, we aimed to quantify different levels of variability in a clinical cohort of 19 NHL patients and identify significant influential factors on CAR-T cell expansion and survival. Finally, we sought to translate the model-estimated T cell expansion capacity into a clinical composite score to propose a cut-off value that allows survival prediction leveraging but not requiring the use of the model.

## 2. Materials and Methods

### 2.1. Patients and Treatment

Our clinical data contained a cohort of 24 adult patients with relapsed or refractory large B cell lymphoma treated with standard of care, axicabtagene ciloleucel, at MD Anderson Cancer Center, Houston, Texas, USA. The study was approved by the MD Anderson Cancer Center’s institutional review board and conducted in accordance with the principles of the Declaration of Helsinki. The 24 patients had different subtypes of non-Hodgkin lymphoma, such as diffuse large B cell lymphoma, primary mediastinal B cell lymphoma and transformed follicular lymphoma. Prior to CAR-T cell infusion on day 0, patients received a low-dose lymphodepleting chemotherapy consisting of fludarabine (30 mg·m^−2^ body surface area per day) and cyclophosphamide (500 mg·m^−2^ body surface area per day) on days −5, −4, and −3. Further patient demographics and clinical characteristics prior to lymphodepleting chemotherapy are shown in detail in Supplementary Table S1. All patients received a single intravenous infusion of axicabtagene ciloleucel at a target dose of 2 × 10^6^ CAR-T cell·kg^−1^ body weight. Of the 24 patients, we excluded five patients for the model analysis. Amongst these, one patient had no active disease during the whole study, for two patients no baseline metabolic tumor volume measurement was available, and for two patients, the staining steps during flow cytometry to determine CAR-T cell concentrations failed.

### 2.2. Tumor Size Measurements and Endpoint Assessment

Metabolic tumor volumes were assessed at baseline, after one month and three months after CAR-T cell infusion using fluorodeoxyglucose positron emission tomography computed tomography (PET-CT). Anti-tumor responses were assessed according to the 2014 Lugano classification [27]. Progression-free survival (PFS) was defined as the time from the start of the axicabtagene ciloleucel infusion to progression of disease, death, or last follow-up (whichever occurred first). Overall survival (OS) was defined as the time from the start of the axicabtagene ciloleucel infusion to death or last follow-up. Patients who were lost to follow-up were from overseas or other US states and did not return to MD Anderson Cancer Center for further monitoring.

### 2.3. CAR-T Cell Sampling, Detection, and Quantification

#### 2.3.1. Sample Collection

Peripheral blood mononuclear cells (PBMCs) were isolated from blood samples by density gradient separation at days 7, 14, and 25–28 after CAR-T cell infusion and cryopreserved for batched analysis.

#### 2.3.2. Cell-Free DNA Real-Time PCR

We used quantitative polymerase chain reaction (qPCR) to determine the DNA copy number of the CAR-T product in patient plasma samples (cell-free DNA, cfDNA). We used appropriate primers (FW—GGATTCGCCAGCCTCCAC; REV—AAACTTGGCTCTTGGAGTTGT) and an endogenous probe (56—FAM/TCCCAGCCA/ZEN/ CTCCAGACCCTT/3IABkFQ/) that correctly identified the chimeric region of CD19-CAR T product in CAR-T-transduced T cells. The DNA copy number from each mg of unknown cfDNA sample was determined using a 10-fold standard curve in which the value corresponding to the DNA copy number was calculated using regression analysis.

#### 2.3.3. Flow Cytometry

PBMC were processed immediately after thawing; the cells were counted, analyzed for viability, and stained using fluorescently labeled antibodies for 30 min (we used antibody concentrations according to the manufacture’s recommendations) at room temperature. We used two antibody panels to measure the phenotype and activation of CAR-T CD4 and CD8 fractions. All cells were pre-treated with an anti-Fc block (130-059-901, Miltenyi Biotec, Bergisch Gladbach, Germany) and Aqua Live/Dead exclusion dye (L34957, Life Technologies, Carlsbad, CA, USA). The list of antibodies in each staining panel is as follows (the clone or reference number followed by the manufacturer’s name is annotated in parenthesis): Cocktail 1—OX40 FITC (Cat No. 555837), anti-CAR T PE (KIP-1, BD Biosciences), ICOS PE-TR (C398.4A, BD Biosciences), CD127 PerCP Cy5.5 (HIL-7R-M21, BD Biosciences, San Jose, CA, USA), CD69 APC (Cat No. 555533, BD Biosciences), CD28 APC-H7 (CD28.2, BD Biosciences), CD4 AF700 (RPA-T4, BD Biosciences), 4-1BB BV421 (4B4-1, BD Biosciences), CD14 BV605 (M5E2, BD Biosciences), CD8 BV650 (RPA-T8, BD Biosciences), CD3 BV711 (UCHT1, BD Biosciences), and PD-1 BV786 (EH12.1, BD Biosciences); and Cocktail 2—CD45RA FITC (Cat No. 555488, BD Biosciences), anti-CAR T PE (KIP-1, BD Biosciences), CD4 PE-TR (RPA-T4, BD Biosciences), CD16 PE-CY7 (3G8, BD Biosciences), CCR7 PerCP Cy5.5 (150503, BD Biosciences), CD25 APC-H7 (M-A251, BD Biosciences), CD27 AF700 (O323, Biolegend, Santiago, CA, USA), CD38 BV605 (HB7, BD Biosciences), CD8 BV650 (RPA-T8, BD Biosciences), CD3 BV711 (UCHT1, BD Biosciences), and CD56 BV786 (NCAM16.2, BD Biosciences). The acquisition of cytometric events varied and depended on the number and the viability of the PBMCs (this number fluctuated from 2 × 10^5^ to 1 × 10^6^ cells). We used a 12-color multiparametric approach using a 3-laser FACS Fortessa Cytometer (BD Biosciences, San Jose, CA, USA). We established a compensation matrix using the DiVa 6.1.1 software with the acquisition of single staining controls. We analyzed FCS files using FlowJo (BD Biosciences, San Jose, CA, USA). We plotted the total events on an SSC vs. FSC quadrant, and we excluded the doublets by gating out the cells on the periphery on the SSC vs. FSC plot (Supplementary Figure S1, top left). We excluded all dead cells by plotting SSC vs. Aqua (Supplementary Figure S1, top middle) and gated on the negative populations (live). We then re-plotted the live events using SSC vs. FSC and gated the lower and upper populations on the right (we did this because activated CAR-T cells appeared larger than normal lymphocytes) to select the lymphocyte populations (Supplementary Figure S1, top right). From the selected gate, we plotted SSC vs. CD3 (Supplementary Figure S1, bottom left); then, from the CD3^+^ populations, we plotted CD3 vs. CAR-T to discriminate all CD3^+^CAR-T^−^ from the CD3^+^CAR-T^+^ cells (Supplementary Figure S1, bottom middle). Using the CD3^+^CAR-T^+^ populations, we then plotted CD4 vs. CD8 to obtain single CD4^+^CAR-T^+^ and CD8^+^CAR-T^+^ populations (Supplementary Figure S1, bottom middle right). To define the phenotype, we plotted the CD4^+^ and CD8^+^ single-stained populations according to their level of expression of CD45RA vs. CCR7, thus, CD45RA^+^CCR7^−^ (T_Eff_), CD45RA^+^CCR7^+^ (T_N_), CD45RA^−^CCR7^+^ (T_CM_), and CD45RA^−^CCR7^−^ (T_EM_) (Supplementary Figure S1, bottom far right). We plotted each single subset vs. every single other marker included in each panel.

#### 2.3.4. Cytokine Measurements

We measured plasma cytokines from CAR-T-infused patients at baseline (day 0), day 4, 7, 9, and 14 using the multiplex assay from Meso Scale Discovery system (Meso Scale 437 Diagnostics; Rockville, MD, USA). We ran 25 µL samples in duplicate to identify Ang-1, Ang-2, EGF, G-CSF, GM-CSF, Granzyme B, GROa (CXCL1), I-TAC (CXCL11), IFN-γ, IL-10, IL-15, IL-1RA, IL-1α, IL-1β, IL-2, IL-2RA, IL-3, IL-5, IL-6, IL-8, IP-10 (CXCL10), M-CSF, MCP-1(CCL2), MIG (CXCL9), MIP-1α(CCL3), TNF-α, VEGF, and VWF, per the manufacturer’s instructions.

### 2.4. Development of the CD19-Specific CAR-T Cell Quantitative Systems Pharmacology Model

We developed the population quantitative systems pharmacology CAR-T cell model by integrating previous knowledge on T cell physiology, adoptive cell therapy, and previously published mathematical immunotherapy models [28,29]. We aimed for our model to be as mechanistic as possible, allowing all parameters to have a physiological meaning while, to enable clinical use, maintaining simplicity following the principle of parsimony. We considered different sources of variability [26] by using a nonlinear mixed-effects modeling approach [30,31].

#### 2.4.1. Nonlinear Mixed-Effects Modeling

A nonlinear mixed-effects model consists of three submodels [32]: the structural submodel, the statistical submodel, and the covariate submodel.

#### 2.4.2. Structural Submodel

The structural submodel (including so called ‘fixed effects’) characterizes the typical concentration–time profile of one or more model species and is described using ordinary differential equations.

#### 2.4.3. Statistical Submodel

The statistical submodel (including so-called ‘random effects’) captures different levels of unexplained variability around the parameters of the structural submodel and the observations. During development of the CAR-T cell quantitative systems pharmacology model, interindividual variability was evaluated on all structural model parameters using exponential interindividual variability models (Equation (1)).

(1) $$\theta_{ik}=\theta_{k}\cdot \;e^{\eta_{ik}}\;\eta_{ik}∼N\;(0,\omega_{k}^{2})$$

with the structural model parameter $\theta_{k}$ and individual model parameter $\theta_{ik}$ for individual *i* = 1,…,N and model parameter *k* = 1,…,N. Individual model parameters on which interindividual variability was implemented were thus assumed to be log-normally distributed with $\eta_{ik}$ values following a normal distribution with mean zero and variance $\omega_{k}^{2}$. To ease interpretation, the interindividual variability was expressed as coefficient of variation, using Equation (2) [32].

(2) $$CV,\%=\sqrt{e^{\omega_{k}^{2}}-1}\;\cdot 100$$

Residual unexplained variability, quantifying the remaining unexplained variability after accounting for interindividual variability [32], was implemented for each model species using a log-transform-both sides approach [30,32] (Equation (3)), equivalent to an additive residual variability model on the log-scale.

(3) $$\ln\left(Y_{ij}^{obs}\right)=\ln\left(f\left(x_{ij},\;\theta_{i}\right)\;\cdot \;e^{\varepsilon_{ij}}\right)\;\varepsilon_{ij}∼\;N\;(0,\sigma_{ij}^{2})\;\;\ln\left(Y_{ij}^{obs}\right)=\ln\left(f\left(x_{ij},\;\theta_{i}\right)\right)+\varepsilon_{ij}\;\mathrm{with}\;\varepsilon_{ij}=\ln(Y_{ij}^{obs})-\ln(Y_{ij}^{pred})$$

In Equation (3), the logarithm of individual observation $Y_{ij}^{obs}$ for individual *i* = 1,...,N and time point *j* = 1,…,N are described as the logarithm of the function of independent design variable $x_{ij}$ (for example sampling times), given the vector $\theta_{i}$ of model parameters for individual *i* = 1,…,N, and the residual unexplained variability parameter $\varepsilon_{ij}$. The residual unexplained variability for individual *i* = 1,…,N at time point *j* = 1,…,N quantifying the deviation between individual model prediction and observation, is implemented as an exponential function and assumed to be normally distributed with mean zero and variance $\sigma_{ij}^{2}$. Applying a log-transformation to both the observation and the model predictions increases the numerical stability of the model, especially if observed concentrations range over several orders of magnitude. Analogous to Equation (2), the residual unexplained variability was expressed as coefficient of variation, using Equation (4).

(4) $$CV,\%=\sqrt{e^{\sigma_{\;}^{2}}-1}\;\cdot 100$$

#### 2.4.4. Covariate Submodel

The covariate submodel (including ‘fixed effects’) aims to explain parts of the interindividual variability identified in the statistical submodel. The covariates (patient-, therapy-, or product-related characteristics) tested for significance during development of the CAR-T cell quantitative systems pharmacology model were pre-selected based on prior literature reports, physiological plausibility, and availability in our clinical study dataset. Exploratory graphical analyses were used to assess the potential size of the covariate effect and guide initial estimate selection.

#### 2.4.5. Model Estimation, Parameter Precision, and Software

In the nonlinear mixed-effects approach ‘fixed-effects-’ and ‘random-effects’ parameters are simultaneously estimated by maximizing the likelihood [33,34]. The objective function value (OFV = −2·ln(likelihood)), a numeric criterion for quality of the model fit, decreases as the quality increases. Model selection for the CAR-T cell quantitative systems pharmacology model was thus based on the objective function value (OFV), parameter precision, and graphical goodness-of-fit evaluation. For the covariate submodel, we evaluated and refined our covariate selection by assessing changes with respect to parameter precision, model stability, individual model predictions, and significance of covariate inclusion using likelihood-ratio tests. At *α* = 0.05, the inclusion of an additional covariate significantly improved model predictions if the OFV decreased by a value of 3.84 points.

If we observed a bi- or multimodal distribution instead of a normal distribution of the individual interindividual variability parameter estimates, suggesting separate subgroups of individuals, we investigated the implementation of a mixture model, allowing to define multiple subpopulations with different sets of typical parameter values. Even though the underlying factor discriminating the subpopulations, i.e., a covariate, might not be known, a mixture model allows to estimate the different parameter values of these subpopulations [35]. During model estimation including a mixture model, the typical values for the model parameters are estimated for each subpopulation. Furthermore, the proportion of individuals in each subpopulation and the most likely subpopulation for each individual are estimated.

To assess parameter uncertainty using standard errors, sampling importance resampling (SIR) [36], using the NONMEM generated covariance matrix to define a proposal distribution, was performed. More specifically, five iterations with 1000, 1000, 1000, 2000, and 2000 samples and 200, 400, 500, 1000, and 1000 resamples, respectively, were chosen. For easier interpretation, standard errors were reported in relation to the parameter estimate [30] as relative standard errors (RSEs). In general, RSEs ≤ 30% for fixed-effects parameters and ≤ 50% for random-effects parameters are considered adequate [30], however, higher RSEs might be acceptable based on the type of analysis, overall relevance of the parameter, and size of the dataset. All modeling activities were performed using the software NONMEM^®^ Version 7.4.3 (ICON Development Solutions, Ellicott City, MD, USA ), called through Perl speaks NONMEM (PsN) Version 3.6.2 [37], using the modeling workbench Pirana Version 2.9.7 (Certara, Princeton, NJ, USA) [38]. For parameter estimation, First-Order Conditional Estimation with Interaction was used. Pre- and post-processing and model evaluation were performed using R Version 3.5.1 (https://www.R-project.org/ (accessed on 12 January 2021)) accessed through RStudio Version 1.2.1184 (http://www.rstudio.com/ (accessed on 12 January 2021)), using packages plyr, dplyr, Xpose4, ggplot2, and scales.

### 2.5. Characterization of Patients in Different Model-Defined (Sub)Populations

During development of the CAR-T cell quantitative systems pharmacology model, two model-defined (sub)populations were identified based on their parameter values: a reference expansion population and a low expansion subpopulation. We investigated differences between both populations, which could potentially explain the observed differences in the model parameters. Continuous variables, i.e., baseline metabolic tumor volume and patient age, as well as frequencies of categorical covariates, i.e., disease type, previous/no previous ASCT and patient sex, were compared. Furthermore, observed and model-predicted cell kinetic parameters (i) maximum concentration of all CAR-T cells (C_max_), (ii) time at maximum concentration of all CAR-T cells (T_max_), (iii) area under the concentration–time curve from day 0 to day 28 (AUC_0–28d_) of all CAR-T cells, and (iv) the ratio of C_max_ of all CAR-T cells over baseline metabolic tumor volume, as a possible predictor for a good prognosis [39], were compared between both subpopulations. To assess statistical significance of differences between continuous covariate or cell kinetic parameter values, two-sided non-parametric Wilcoxon tests (α = 0.05) using the function ‘compare_means’ of the R package *ggpubr* were performed. The results were visualized with box-whisker plots for continuous covariates and bar plots for categorical covariates using R package *ggplot2*.

### 2.6. Clinical Endpoints in Patients of Different Model-Defined (Sub)Populations

To compare clinical endpoints in different patient (sub)populations, Kaplan–Meier curves were generated and stratified for the respective variable (e.g., expansion subpopulation or previous/no previous ASCT) using the R packages survival and survminer. Log-rank tests (α = 0.05) were used to assess if there was a significant difference between the curves. If we compared several strata within one plot, we performed pairwise comparisons in addition to the global log-rank test. A possible correlation between model parameter V_max1_ and clinical composite score (CCS) C_max_/baseline metabolic tumor volume was assessed for each CAR-T cell phenotype and the sum of CAR-T cell phenotypes (T_all_) using Pearson correlation tests through the ‘ggscatter’ function in the R package ggpubr. For the CAR-T cell phenotype for which the CCS showed the highest correlation with V_max1_, we assessed an optimal cut-off value of the CCS for detecting patients in the low expansion subpopulation, by performing a receiver operating characteristic (ROC) curve analysis using the R packages cutpointr and pROC [40]. Next, we performed univariate cox-proportional hazard analyses using the R package survival to assess if the identified CCS cut-off value for the chosen CAR-T cell phenotype was a significant predictor for survival. We tested and confirmed the proportional hazard assumption using the function ‘cox.zph’ in the R survival package. Finally, the correlation between the CCS using flow cytometry and the CCS using qPCR was quantified using a Pearson-correlation test. The CCS_qPCR_ was compared between reference expansion population and low expansion subpopulation using a two-sided Wilcoxon test. Two additional previously digitized datasets [41], reporting C_max_ (assessed by qPCR) and baseline tumor burden in CLL [42] and MM [43] patients, were digitized (Supplementary Figure S2). Next, CCS_qPCR_ were computed and the differences between patients with CR/PR and PD/NR assessed using two-sided Wilcoxon tests.

## 3. Results

### 3.1. The CD19-Specific CAR-T Cell Quantitative Systems Pharmacology Model

Based on previous reports regarding the impact of CAR-T cell phenotype composition on CAR-T cell in vivo expansion and persistence [44], we chose to describe each CAR-T cell phenotype measured in our clinical cohort (T_N_, T_CM_, T_EM_, and T_Eff_) as individual species. As a fifth species, we included CD19^+^ metabolic tumor volume as a pharmacodynamic component and a key driver of CAR-T cell expansion. To jointly describe typical profiles of concentrations of CAR-T cell phenotypes and CD19^+^ tumor volume across time and different layers of variability, we used nonlinear mixed-effects modeling.

#### 3.1.1. Structural Submodel

**Figure 1:**
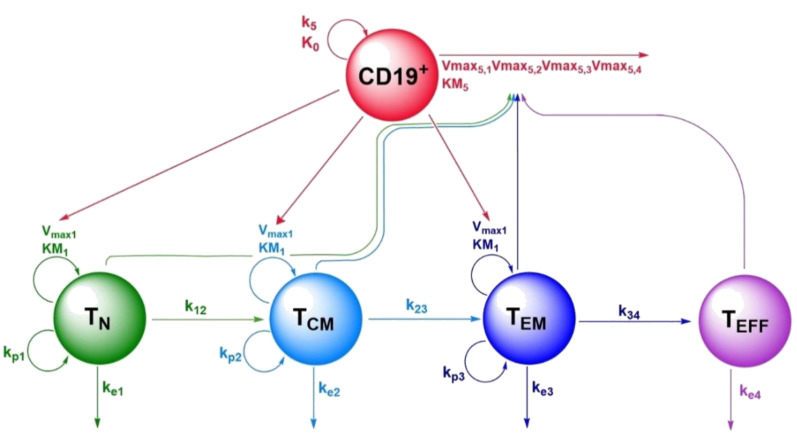
Schematic representation of the CD19-specific CAR-T cell population quantitative systems pharmacology model, describing kinetics and dynamics of the four CAR-T cell phenotypes and CD19^+^ tumor metabolic tumor volume. Legend: Non-red arrows pointing to the right describe differentiation processes. Non-red downward arrows represent cell death processes. Circular arrows represent proliferation processes. The red arrow pointing to the right represents CD19^+^ tumor death. Arrows pointing to parameter names indicate a positive impact on this parameter by the species of which the arrow is originating from. Abbreviations—T_N_: naïve CAR-T cells; T_CM_: central memory CAR-T cells; T_EM_: effector memory CAR-T cells; T_Eff_: effector CAR-T cells; CD19^+^: CD19^+^ metabolic tumor volume; k_12_: rate constant for differentiation of T_N_ to T_CM_; k_23_: rate constant for differentiation of T_CM_ to T_EM_; k_34_: rate constant for differentiation of T_EM_ to T_Eff_; ke_1_: death rate constant for T_N_; ke_2_: death rate constant for T_CM_; ke_3_: death rate constant for T_EM_; ke_4_: death rate constant for T_Eff_; kp_1_: homeostatic proliferation rate constant for T_N_; kp_2_: homeostatic proliferation rate constant for T_CM_; kp_3_: homeostatic proliferation rate constant for T_EM_; V_max1_: maximum expansion rate per mL tumor volume of T_N,_ T_CM_ and T_EM_ upon tumor contact; KM_1_: T_N_, T_CM_ and T_EM_ concentration at half-maximum expansion of T_N_, T_CM_ and T_EM_; V_max5,1_: maximum killing rate of metabolic tumor volume by T_N_; V_max5,2_ maximum killing rate of metabolic tumor volume by T_CM_; V_max5,3_: maximum killing rate of metabolic tumor volume by T_EM_; V_max5,4_: maximum killing rate of metabolic tumor volume by T_Eff_; KM_5_: metabolic tumor volume at half-maximum killing rate; k_5_: proliferation rate constant of metabolic tumor volume; K_0_: maximum tumor volume observable (tumor carrying capacity).

The structural submodel of the nonlinear mixed-effects quantitative systems pharmacology model consisted of five species: naïve CAR-T cells (T_N_), central memory CAR-T cells (T_CM_), effector memory CAR-T cells (T_EM_), terminally differentiated effector CAR-T cells (T_Eff_), and CD19^+^ metabolic tumor volume (CD19^+^) (Figure 1). For the nonlinear processes describing T cell expansion upon tumor contact and tumor killing upon CAR-T cell contact, different functional forms were explored. While the numerator always consisted of the product $V_{\max,x}\cdot \mathrm{CD}19^{+}\cdot T_{\mathrm{cell}}$, we tested three versions for the denominator, limiting the maximum expansion either by the respective T cell concentration, the metabolic tumor volume or both. For both terms, the form which described the data best was selected.

We described the lineage relationship of the four CAR-T cell phenotypes according to the progressive differentiation model, which postulates the differentiation of naïve T cells via memory T cells to terminally differentiated effector T cells [45,46].

##### Naïve CAR-T Cells (T_N_)

Upon contact with CD19^+^ tumor cells, we modeled T_N_ to expand with maximum expansion rate per mL tumor volume V_max1_ and naïve T cell concentration at half-maximum expansion rate KM_1_ (1.13 cells·µL^−1^, relative standard error (RSE): 22%). Independent of expansion upon tumor cell contact, T_N_ undergo homeostatic proliferation with a first-order rate constant k_p1_ (0.0005·day^−1^) [47]. Furthermore, we described T_N_ to differentiate into T_CM_ with the first-order rate constant k_12_ (0.140·day^−1^, RSE: 9%) and undergo apoptosis corresponding to a typical lifespan of 1/k_e1_ (1/0.0104·day^−1^ = 96 days, RSE: 13%) days. The resulting typical profile for T_N_ is given by Equation (5).

(5) $$\frac{d}{\mathrm{dt}}T_{N}=\frac{V_{\max1}\cdot \;\mathrm{CD}19^{+}\cdot {\;T}_{N}\;}{{\mathrm{KM}}_{1}+{\;T}_{N}}+{\;\mathrm{kp}}_{1}\cdot T_{N}-k_{12}\cdot T_{N}-{\;k}_{e1}\cdot T_{N}$$

##### Central Memory CAR-T Cells (T_CM_)

Analogous to T_N_, we modeled T_CM_ to expand upon tumor contact with the same Michaelis–Menten parameters V_max1_ and KM_1_ and to undergo homeostatic proliferation with the rate constant kp_2_ (0.007·day^−1^) [47]. Moreover, we described concentrations of T_CM_ to increase by differentiation of T_N_ with the rate constant k_12_. In line with the progressive differentiation model, concentrations of T_CM_ were described to decrease due to differentiation into T_EM_ with the rate constant k_23_ (0.191·day^−1^, RSE: 11%) or apoptosis after a typical lifespan of 1/k_e2_ (1/0.0104·day^−1^ = 96 days, RSE: 13%) days. The resulting typical profile of T_CM_ is given by Equation (6).

(6) $$\frac{d}{\mathrm{dt}}T_{\mathrm{CM}}=\frac{V_{\max1}\;\cdot \;\mathrm{CD}19^{+}\cdot {\;T}_{\mathrm{CM}}}{{\mathrm{KM}}_{1}+{\;T}_{\mathrm{CM}}}+{\;\mathrm{kp}}_{2}\cdot {\;T}_{\mathrm{CM}}+k_{12}\cdot T_{N}-k_{23}\cdot {\;T}_{\mathrm{CM}}-{\;k}_{e2}\cdot {\;T}_{\mathrm{CM}}$$

##### Effector Memory CAR-T Cells (T_EM_)

Analogous to T_N_ and T_CM_, we modeled T_EM_ cells to expand upon tumor contact in a nonlinear process with the parameters V_max1_ and KM_1_. In addition, T_EM_ cells were described to undergo linear homeostatic proliferation with the rate constant kp_3_ (0.007·day^−1^) [47] and to be formed via differentiation of T_CM_ with the rate constant k_23_. Moreover, we described T_EM_ to differentiate into T_Eff_ with the rate constant k_34_ (0.355·day^−1^, RSE: 13%) and to undergo apoptosis after a typical lifespan of 1/ke_3_ (1/0.0104·day^−1^ = 96 days, RSE: 13%) days. The resulting typical profile of T_EM_ is given by Equation (7):

(7) $$\frac{d}{\mathrm{dt}}T_{\mathrm{EM}}=\frac{V_{\max1}\;\cdot \;\mathrm{CD}19^{+}\cdot {\;T}_{\mathrm{EM}}}{{\mathrm{KM}}_{1}+{\;T}_{\mathrm{EM}}}+{\;\mathrm{kp}}_{3}\cdot {\;T}_{\mathrm{EM}}+k_{23}\cdot T_{\mathrm{CM}}-k_{34}\cdot {\;T}_{\mathrm{EM}}-{\;k}_{e3}\cdot {\;T}_{\mathrm{EM}}$$

##### Terminally Differentiated Effector CAR-T Cells (T_Eff_)

In contrast to naïve and memory T cells, we considered T_Eff_ cells unable to expand further in response to tumor contact or as homeostatic proliferation. We still considered them to be formed by differentiation of T_EM_ with the rate constant k_34_. In line with previous findings [21,48], as shown in the high estimate for ke_4_, we approximated that a high fraction of T_Eff_ will die each day (0.518·day^−1^, RSE: 13%). The resulting typical profile of T_Eff_ is given by Equation (8).

(8) $$\frac{d}{\mathrm{dt}}T_{\mathrm{Eff}}={\;k}_{34}\cdot T_{\mathrm{EM}}-{\;k}_{e4}\cdot {\;T}_{\mathrm{Eff}}$$

##### CD19^+^ Metabolic Tumor Volume (CD19^+^)

We modeled CD19^+^ metabolic tumor volume growth with a logistic growth function [28] with growth parameter k_5_ (0.0023 day^−1^) and carrying capacity K_0_ (5000 mL), which represents the highest metabolic tumor volume observable (Equation (9)). Tumor cell killing by the different CAR-T cell phenotypes was adapted from a previously published tumor immune reaction mathematical model [28] as a nonlinear process with maximum killing rate V_max5,x_ (with *x* = 1−4 representing the four CAR-T cell phenotypes in the order naïve, central memory, effector memory and effector), and metabolic tumor volume at half-maximum killing rate KM_5_ (276 mL, RSE: 33%). While for parameter V_max5,2_, the maximum killing rate for T_CM_ was estimated (4.04 mL·day^−1^·(cells·µL^−1^)^−1^, RSE: 39%), the maximum killing rates for the other T cell phenotypes were fixed based on the estimate for V_max5,2_ and fractional changes in killing capacities extracted from a digitized plot showing in vitro killing capacities of different CAR-T cell phenotypes [49].

(9) $$\begin{array}{cc} \frac{d}{\mathrm{dt}}\mathrm{CD}19^{+}=k_{5}\cdot & (1-\frac{\mathrm{CD}19^{+}}{K_{0}})\cdot \mathrm{CD}19^{+}\;\frac{-\;V_{\max5,1}\;\cdot \;T_{N}\;\cdot \;\mathrm{CD}19^{+}}{{\mathrm{KM}}_{5}+\mathrm{CD}19^{+}\;}\\ & -\;\frac{V_{\max5,2}\;\cdot \;T_{\mathrm{CM}}\;\cdot \;\mathrm{CD}19^{+}}{{\mathrm{KM}}_{5}+\mathrm{CD}19^{+}\;}-\frac{V_{\max5,3}\;\cdot \;T_{\mathrm{EM}}\cdot \;\mathrm{CD}19^{+}\;}{{\mathrm{KM}}_{5}+\mathrm{CD}19^{+}\;}\\ & -\;\frac{V_{\max5,4}\;\cdot \;T_{\mathrm{Eff}}\cdot \;\mathrm{CD}19^{+}\;}{{\mathrm{KM}}_{5}+\mathrm{CD}19^{+}} \end{array}$$

#### 3.1.2. Statistical Submodel

We implemented interindividual variability parameters on V_max1_ (446% CV) and V_max5,2_ (307% CV) using Equation (1)_._ The implementation of interindividual variability parameters on other structural submodel parameters was not supported by the dataset. Applying Equations (5)–(9) with the estimated parameter values to the measured concentrations of (i) the four CAR-T cell phenotypes and (ii) metabolic tumor volumes in our clinical dataset (*n* = 19 patients, Table S1), population and individual model predictions were in line with observed values for the majority of individuals. However, for some patients, typical predictions exceeded the measured T cell concentrations by up to 270-fold. A common feature of these patients was that T cells failed to expand as expected based on the high baseline tumor burden. Furthermore, we observed a bimodal distribution of individual V_max1.base_ estimates. Based on this observation, we used a mixture model to investigate the presence of two subpopulations with separate estimates for V_max1,base_. We precisely estimated 20% (RSE: 11%) (*n* = 4) of patients to belong to a low expansion subpopulation with a significantly reduced (by 92%, *p* = 0.0043) typical value for V_max1,base_ (V_max1,base,low_: 0.000700 (cells·µL^−1^)·day^−1^·mL^−1^, RSE: 17%) compared to the reference population (V_max1,base,ref_: 0.00846 (cells·µ^−1^)·day^−1^·mL^−1^, RSE: 36%). The mixture model’s implementation significantly improved and aligned typical and individual predictions for the low expansion subpopulation (Supplementary Figure S3).

#### 3.1.3. Covariate Submodel

We pre-selected a previous ASCT, the ratio of CD4^+^ to CD8^+^ CAR-T cells on day seven and the concentrations of IL-2 and IL-15 for evaluation as covariates on model parameter V_max1,base_. Furthermore, we pre-selected tumor type and concentrations of Granzyme-B, TNFα, and IFN-γ on day seven for evaluation on model parameter V_max5,2_. Ratios of CD4^+^ over CD8^+^ CAR-T cells and cytokine concentrations were additionally available at baseline and on days four, nine, 14 and 28. However, measurements were not available for all patients at all time points. As measurements were available for 18 of 19 patients on day seven, we chose this time point for implementation.

We identified two significant covariates on the baseline maximum expansion rates V_max1,base,ref_ and V_max1,base,low_. A previous ASCT was incorporated as dichotomous covariate (ASCT = 0: no previous ASCT, ASCT = 1: previous ASCT) and the change in V_max1,base_ due to a previous ASCT was implemented using a fractional change model (ASCT_Vmax1_: 2.53, RSE: 31%, translating into a 3.53-fold higher V_max1,base_ value in patients with a previous ASCT). Of note, the covariate effect for a previous ASCT was estimated for all patients simultaneously using their respective V_max1,base_ value (V_max1,base,ref_ or V_max1,base,low_) instead of estimating separate effects of a previous ASCT for V_max1,base,ref_ and V_max1,base,low_. The second covariate, the ratio of CD4^+^/CD8^+^ CAR-T cells at day seven (${\mathrm{CAR}}^{+}\mathrm{CD}4/\mathrm{CD}8_{\mathrm{day}7})$, was implemented on V_max1,base,ref_ using a power function. An increasing ratio of CD4/CD8^+^ CAR-T cells at day seven was associated with a moderate decrease in V_max1,base,ref_ (CD4/CD8_exp_: −0.385, RSE: 45%). As for the low expansion subpopulation, an exploratory graphical analysis showed that only a previous ASCT but not the ratio of CD4^+^/CD8^+^ CAR-T cells at day seven was influential on the baseline maximum expansion capacity V_max1,base,low_ (Supplementary Figure S4) and only a previous ASCT remained as covariate on V_max1,base,low_. There was no significant relationship between other possible covariates and V_max1,base_. The final equations for V_max1_ applicable to the reference and low expansion (sub)populations are shown in Equations (10) and (11), respectively. In these equations, V_max1,ref_ and V_max1,low_ are the maximum expansion rates per mL metabolic tumor volume in the reference and the low expansion (sub)population, respectively, based on (i) the typical maximum expansion rates per mL metabolic tumor volume in the reference population $\left(V_{\max1,\mathrm{base},\mathrm{ref}}\right)$ or the low expansion subpopulation $\left(V_{\max1,\mathrm{base},\mathrm{low}}\right)$, (ii) the fractional change in $V_{\max1,\mathrm{base},\mathrm{ref}}$ or $V_{\max1,\mathrm{base},\mathrm{low}}$ due to a previous ASCT (${\mathrm{ASCT}}_{\mathrm{Vmax}1}$), and (iii) for the reference population the change in $V_{\max1,\mathrm{base},\mathrm{ref}}$ based on the measured ratio of CD4^+^/CD8^+^ CAR-T cells on day 7 $({\mathrm{CAR}}^{+}\mathrm{CD}4/\mathrm{CD}8_{\mathrm{day}7}$) to the power of model-estimated exponent $\mathrm{CD}4/\mathrm{CD}8_{\exp}$.

(10) $$V_{\max1,\mathrm{ref}}=V_{\max1,\mathrm{base},\mathrm{ref}}\cdot \left(1+{\mathrm{ASCT}}_{\mathrm{Vmax}1}\cdot \mathrm{ASCT}\right)\cdot \;{\left({\mathrm{CAR}}^{+}\mathrm{CD}4/\mathrm{CD}8_{\mathrm{day}7}\right)}^{\mathrm{CD}4/\mathrm{CD}8_{\exp}}$$

(11) $$V_{\max1,\mathrm{low}}=V_{\max1,\mathrm{base},\mathrm{low}}\cdot \left(1+{\mathrm{ASCT}}_{\mathrm{Vmax}1}\cdot \mathrm{ASCT}\right)$$

**Figure 2:**
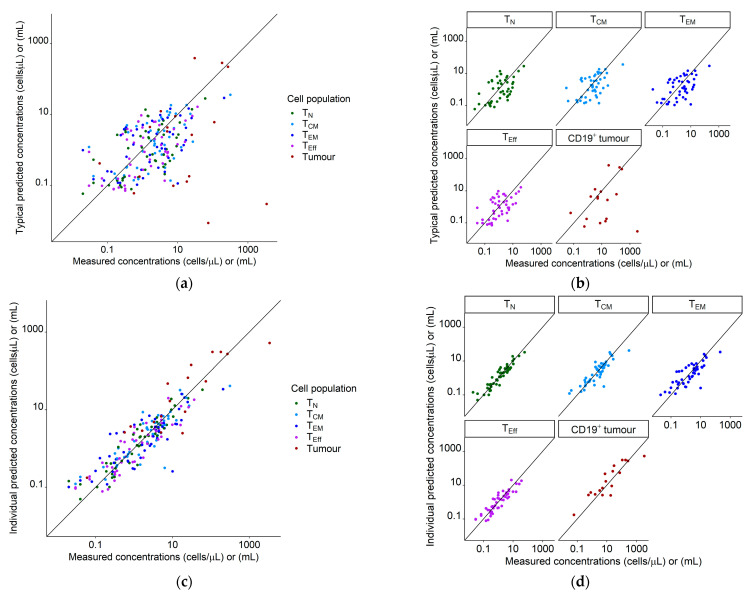
Goodness of fit plots for the population quantitative systems pharmacology model using our clinical dataset of 19 patients. Legend: (**a**) Typical predictions (not considering interindividual variability) vs. measured concentrations/volumes for all species; (**b**) typical predictions (not considering interindividual variability) vs. measured concentrations/volumes, stratified for species; (**c**) individual predictions vs. measured concentrations for all species; (**d**) individual predictions vs. measured concentrations, stratified for species. Diagonal line: Line of identity. Tumor measurements marked as a complete response were set to a value of 0 mL and are not shown. Abbreviations—T_N_: naïve T cells, T_CM_: central memory T cells, T_EM_: effector memory T cells, T_Eff_: effector T cells, CD19^+^ tumor: CD19^+^ metabolic tumor volume.

**Figure 3:**
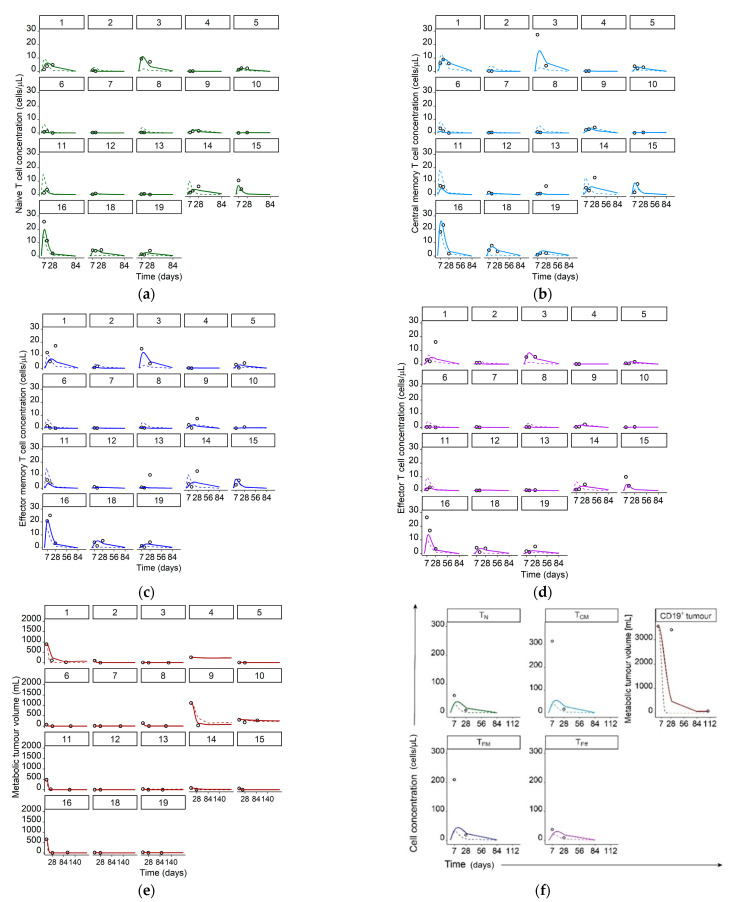
Measured T cell concentrations/metabolic tumor volumes (data points) and simulated typical (dashed lines) and individual (solid lines) model predictions for individual patients 1–16, and 18–19 in panels a–e and, as concentrations/volumes of all species were significantly higher, for patient 17 in separate panel (**f**). Legend: (**a**) Naïve CAR-T cells; (**b**) Central memory CAR-T cells; (**c**) Effector memory CAR-T cells; (**d**) Effector CAR-T cells; (**e**) CD19^+^ metabolic tumor volume; (**f**) Concentrations of naive, central memory, effector memory, and effector CAR-T cells as well as CD19^+^ metabolic tumor volume for patient 17. Abbreviations—T_N_: naïve CAR-T cells, T_CM_: central memory CAR-T cells, T_EM_: effector memory CAR-T cells, T_Eff_: effector CAR-T cells, CD19^+^ tumor: CD19^+^ metabolic tumor volume.

Upon implementation of mixture model and covariates, the interindividual variability in V_max1,base,ref_ was substantially reduced from 446% to 150% (RSE: 19%) CV. The interindividual variability in V_max1,base,low_ was negligible and not included in the model. Final model predictions for concentration–time profiles of all T cell phenotypes and metabolic tumor volume corresponded well with the observations as shown in goodness of fit plots (Figure 2) and observations overlaid with model predictions (Figure 3).

Both Figure 2 and Figure 3 show individual and typical predictions, which are model predictions considering and not considering unexplained interindividual variability, respectively. Differences in typical predictions among individuals arose due to the explained interindividual variability already incorporated in the ‘fixed-effects’ parameters, such as different metabolic tumor volumes as model input or covariate effects. The adequate alignment of typical and individual predictions with the measured concentrations indicated no missed important process in the structural model and/or no missing covariate. After accounting for interindividual variability we quantified the remaining unexplained variability with residual unexplained variability parameters (CV: 59% (RSE: 11%), 86% (RSE: 9%), 120% (RSE: 9%), 71% (RSE: 10%), and 115% (RSE: 12%) for T_N_, T_CM_, T_EM_, T_Eff_, and CD19^+^ tumor, respectively).

#### 3.1.4. Model Estimation and Parameter Precision

No information regarding initial CAR-T cell concentrations in the individual infusion products was available. We mainly focused on CAR-T cell expansion, which is primarily influenced by the tumor burden and the intrinsic CAR-T cell expansion capacity. Thus, we decided to discount the initial distribution phase and assume a low dose of 0.1 cells·µL^−1^ per phenotype as dose. It is plausible to observe this concentration after the initial distribution phase post-infusion. A subsequent sensitivity analysis showed that a ten-fold change of this value had a minor impact on the time of maximum T cell concentration but not on the maximum concentration itself (Supplementary Figure S5), which is in line with previously published data [13]. In addition, our model’s ability to describe the observed data well using the imputed doses supports previous findings of CAR-T cell doses not being predictive of expansion or response [3,18,41].

Most of the parameter values were estimated based on clinical data of CAR-T cell concentrations and metabolic tumor volume. However, using our clinical data, not all model parameters could be estimated: As our data only contained measurements of metabolic tumor volume in the presence of CAR-T cells, parameters k_5_ and K_0_, which describe undisturbed tumor growth and the largest tumor volume observable, were not identifiable and fixed to plausible values. Similarly, homeostatic proliferation rate constants were set to literature values [47] as we performed our CAR-T cell concentration measurements during the rapid expansion phase. As the proliferation in response to target engagement is much faster than homeostatic proliferation, homeostatic proliferation rate constants were unidentifiable. Finally, death rate constants for T_N_, T_CM_ and T_EM_ were unidentifiable and fixed based on the estimated death rate constant for T_Eff_ and the relationship between death rate constants of short- and long-lived cells (2%), according to Stein et al. [21]. Final model parameter values are shown in Table 1.

### 3.2. Characterization of Patients in Different Model-Defined (Sub)Populations

**Figure 4:**
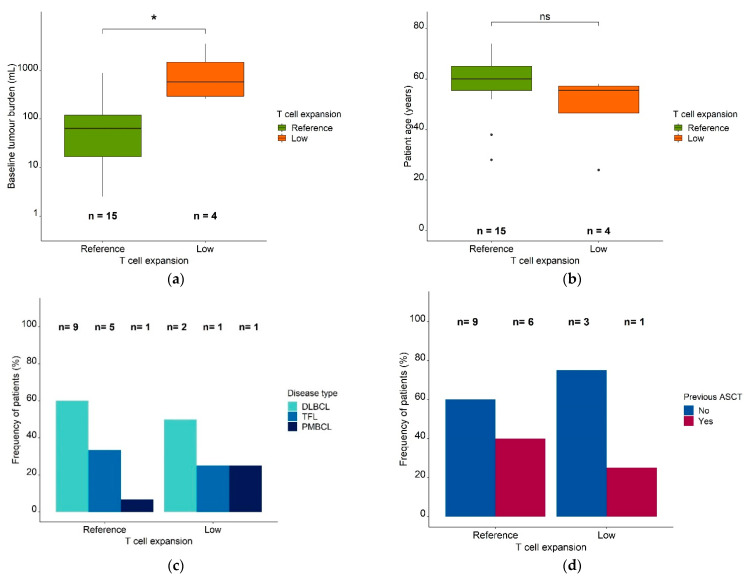
Patient characteristics in the reference expansion population and the low expansion subpopulation. Legend: (**a**) Baseline metabolic tumor volume (mL) in the reference expansion population and the low expansion subpopulation. (**b**) Patient age in the reference expansion population and the low expansion subpopulation. Boxes: interquartile range (IQR) including median; whiskers: range from hinge to lowest/highest value within 1.5 IQR; points: data outside whisker. (**c**) Frequency of patients in the reference expansion population and the low expansion subpopulation, stratified for disease type. (**d**) Frequency of patients in the reference expansion population and the low expansion subpopulation, stratified for a previous ASCT. Abbreviations—ASCT: autologous stem cell transplantation, DLBCL: diffuse large B cell lymphoma, PMBCL: primary mediastinal B cell lymphoma, TFL: transformed follicular lymphoma, *: *p* ≤ 0.05, ns: *p* > 0.05.

To explore reasons for the differences between the reference (*n* = 15) and low expansion (*n* = 4) (sub)populations, we compared clinical characteristics amongst patients in the respective groups: the low expansion subpopulation showed a significantly higher baseline metabolic tumor volume (median: 712 mL, range: 264 mL–3555 mL vs. median: 64.1 mL, range: 2.54 mL–894 mL, *p* = 0.019, Figure 4a).

The median patient age (48 years vs. 58 years) (Figure 4b) and the number of previous therapies (5 vs. 5) were similar in both (sub)populations. In addition, males and females were similarly distributed in the reference population and the low expansion subpopulation [females: 83% and 17%, respectively; males: 77% and 23%, respectively]. The patients in the reference population (*n* = 15) had diffuse large B cell lymphoma (DLBCL; 60% [*n* = 9]), transformed follicular lymphoma (TFL; 33.3% [*n* = 5]), and primary mediastinal lymphoma (PMBCL; 6.67% [*n* = 1]). Around 50% (*n* = 2) of the low expansion subpopulation (*n* = 4) had DLBCL, 25% (*n* = 1) had TFL, and 25% (*n* = 1) had PMBCL (Figure 4c). While 40% (*n* = 6) of patients in the reference expansion population (*n* = 15) had received a previous ASCT, only 25% (*n* = 1) of the patients in the low expansion subpopulation (*n* = 4) had (Figure 4d).

**Figure 5:**
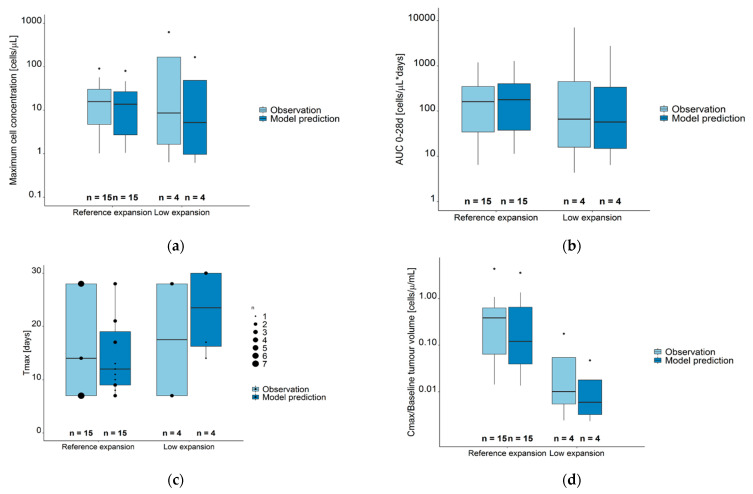
Observed (light blue boxes) and model predicted (dark blue boxes) CAR-T cell kinetic parameters for the sum of all CAR-T cell populations. (**a**) Maximum cell concentration (Cmax). (**b**) Area under the concentration–time curve from day 0–28 (AUC_0–28d_). (**c**) Time at maximum concentration (days); data points of differences sizes mark the number of observations/predictions at different time points. (**d**) Cmax/Baseline metabolic tumor volume (cells µL^−1^ mL^−1^). Legend—Boxes: interquartile range (IQR) including median; whiskers: range from hinge to lowest/highest value within 1.5 IQR; points: data outside whisker. Abbreviations—Tmax: time at maximum CAR-T cell concentration; AUC 0–28d: area under the concentration–time curve from days 0–28, Cmax: maximum CAR-T cell concentration.

There was a high agreement between observations and model predictions for the CAR-T cell kinetic parameters (Figure 5, Supplementary Table S2). C_max_ and AUC_0–28d_ were similar in reference and low expansion (sub) populations, while T_max_ were earlier in the reference compared to the low expansion (sub) population. When observed C_max_ values were normalized to baseline metabolic tumor volumes, these ratios were significantly higher in the reference compared to the low expansion subpopulation (*p* = 0.024).

### 3.3. Clinical Endpoints in Different Model-Defined Patient Subpopulations

**Figure 6:**
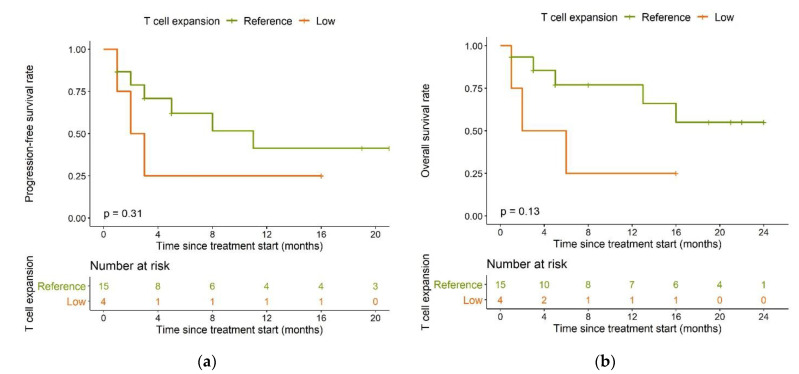
Kaplan–Meier plots for (**a**) progression-free survival and (**b**) overall survival in the reference T cell expansion population (green) and the low expansion subpopulation (orange); log-rank tests.

Compared to the reference population, the low expansion subpopulation had shorter PFS (median: 2.5 months vs. 11 months, *p* = 0.31, Figure 6a) and overall survival (OS) (median: four months vs. not reached, *p* = 0.13, Figure 6b).

**Figure 7:**
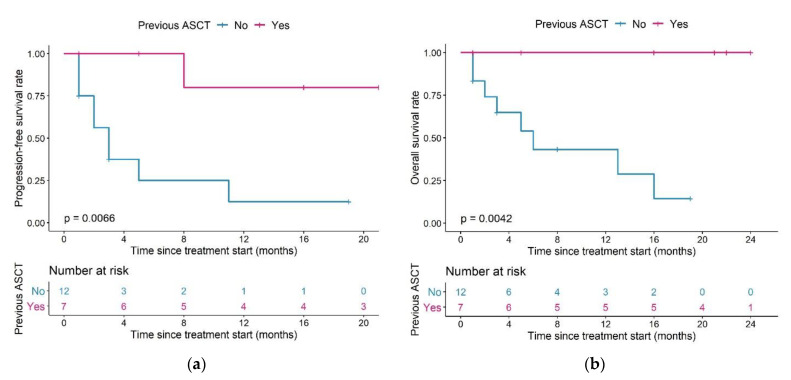
Kaplan–Meier plots for (**a**) progression-free survival and (**b**) overall survival in patients having undergone (magenta) or having or not undergone (blue) a previous ASCT; log-rank tests. Abbreviations—ASCT: autologous stem cell transplantation.

Patients having undergone a previous ASCT (*n* = 7) had a significantly longer PFS compared to patients who had not (*n* = 12) (median PFS: Not reached vs. three months, *p* = 0.0066, Figure 7a) and this difference remained in OS (median OS: Not reached vs. six months, *p* = 0.0042, Figure 7b).

**Figure 8:**
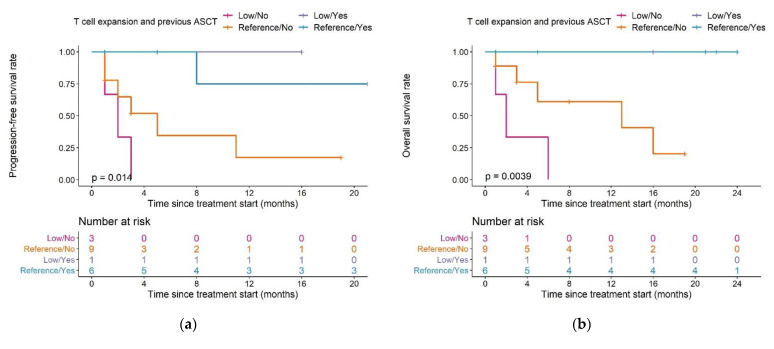
Kaplan–Meier plots for (**a**) progression-free survival and (**b**) overall survival in patients with different combinations of T cell expansion and previous ASCT group; log-rank tests. Pairwise comparisons were performed to assess between which curves there was a significant difference (**a**): significant difference between Reference/Yes and Low/No, *p* = 0.019, (**b**): significant difference between Reference/Yes and Low/No, *p* = 0.026). Legend—Magenta curve: Patients in the low expansion subpopulation who did not undergo a previous ASCT; orange curve: patients in the normal expansion subpopulation who did not undergo a previous ASCT; purple curve: patients in the low expansion subpopulation who underwent a previous ASCT, blue curve: patients in the reference expansion population who underwent a previous ASCT. Abbreviations—ASCT: autologous stem cell transplantation.

We also observed differences in PFS and OS in patients with different combinations of ASCT pre-treatment and T cell expansion capacity (Figure 8a,b): both median PFS and OS in patients in the low expansion subpopulation with no previous ASCT (*n* = 3) were two months.

For patients in the reference expansion population with no previous ASCT (*n* = 9), median PFS was five months, and median OS was 13 months. In patients with a previous ASCT, both median PFS and OS were not reached in both the reference population (*n* = 6) and the low expansion subpopulation (*n* = 1).

All patients who had undergone previous ASCT (*n* = 7) were still alive after a maximum of 24 months of follow-up. Among patients with no previous ASCT, OS was different between patients in the reference or low expansion (sub)population: 44.4% of patients were still alive in the reference population (*n* = 9), compared to 0% of patients in the low expansion subpopulation (*n* = 3).

PFS and OS were significantly different between patients in the reference expansion subpopulation who did (*n* = 6) or did not (*n* = 9) undergo ASCT (median PFS and OS: not reached vs. five months (*p* = 0.039) and not reached vs. 13 months (*p* = 0.02), in patients with or without previous ASCT, respectively).

**Figure 9:**
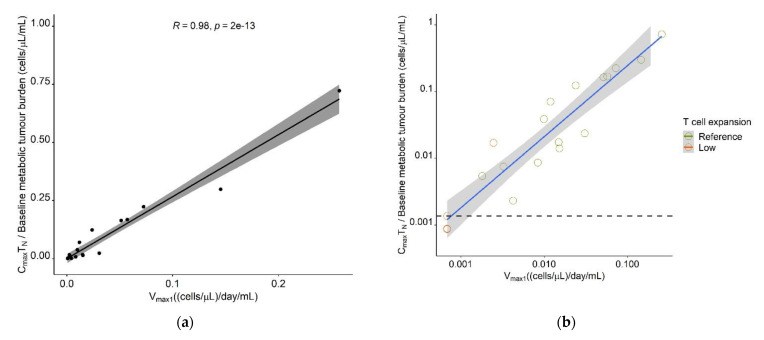

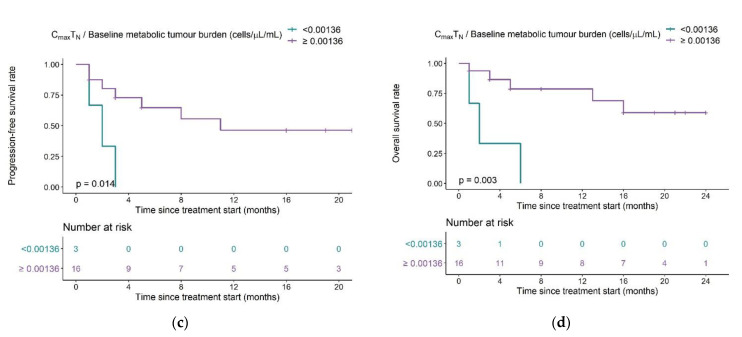
Determination of a clinical composite score (CCS) cut-off value for early response-prediction. (**a**) Correlation between model parameter V_max1_ ((cells·µL^−1^)·day^−1^·mL^−1^) and CCS Maximum naïve CAR-T cell concentration/Metabolic tumor volume at baseline ((cells·µL^−1^) ·mL^−1^). (**b**) Correlation plot as **A** but on a log-log scale and different labeling of the T cell expansion subpopulation. The dashed horizontal line marks the CCS cut-off value most predictive for allocation to the low expansion subpopulation, as determined using ROC analysis. (**c**) Kaplan–Meier probabilities for progression-free survival in patients below or exceeding the determined CCS_TN_ cut-off value. (**d**) Kaplan–Meier plots for overall survival in patients below or exceeding the determined CCS_TN_ cut-off value. Abbreviations—CCS_TN_: clinical composite score for naïve CAR-T cells.

The model-estimated maximum expansion capacity, V_max1_, allowed to identify a patient’s expansion (sub-)population, which was associated with survival; thus, we aimed to determine a cut-off value in this parameter, which would support survival prediction. Furthermore, we aimed to translate V_max1_ into a predictor variable, which would be easily derivable in a clinical setting and leverage, but not require the use of the model. As a measurable clinical composite score (CCS) describing T cell expansion, inspired by a similar concept in anti-PD1 checkpoint blockade [39] and supported by a previous correlative analysis [50], the ratio of observed C_max_ ((cells·µL^−1^))/baseline metabolic tumor volume (mL), denoted in Equation (12), was positively correlated with V_max1_ for all CAR-T cell phenotypes (T_N_: r = 0.98, T_CM_: r = 0.95, T_EM_: r = 0.94, T_EFF_: r = 0.86, T_all_: r = 0.94). As the highest correlation was observed for T_N_ (Figure 9a), the CCS for T_N_ (CCS_TN_) was taken forward as a possible predictor for survival.

(12) $$\mathrm{Clinical}\;\mathrm{composite}\;\mathrm{score}\;\left(\mathrm{CCS}\right)=\frac{\mathrm{Maximum}\;\mathrm{CAR}-T\;\mathrm{cell}\;\mathrm{concentration}}{\mathrm{Baseline}\;\mathrm{metabolic}\;\mathrm{tumour}\;\mathrm{volume}\;}$$

A receiver operator characteristic curve (ROC) analysis determined a CCS_TN_ of 0.00136 (cells·µL^−1^)·mL^−1^ as cut-off value with optimal predictive capability for patients in the low expansion subpopulation (sensitivity: 75%, specificity: 100%; AUC: 91.7%) (Figure 9b and Supplementary Figure S6). The survival analysis, stratified for the proposed cut-off value, showed a clear superiority in survival in patients with a CCS_TN_ ≥ 0.00136 (cells·µL^−1^)·mL^−1^ compared to patients with a CCS below this value (median PFS: 11 months vs. 2 months (*p* = 0.014) and median OS: not reached vs. two months (*p* = 0.003)) (Figure 9c,d). Using a Cox-proportional hazard model, the estimated hazard ratios for PFS and OS in patients with a CCS_TN_ above the proposed threshold were 0.17 (95% CI: 0.037–0.79) (*p* = 0.024) and 0.12 (95% CI: 0.025–0.63) (*p* = 0.012), respectively, suggesting that a CCS_TN_ above the proposed threshold was associated with a 83% reduced risk of progression and a 88% reduced risk of death.

**Figure 10:**
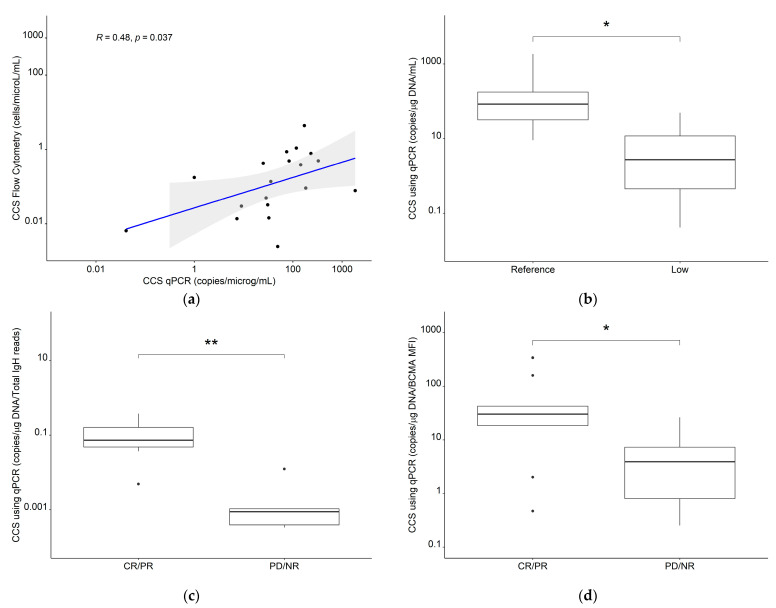
(**a**) Correlation for the clinical composite score for T_all_ using flow cytometry or qPCR and comparisons of the CCS using qPCR between (**b**) reference expansion population and low expansion subpopulation in our clinical dataset, (**c**) CR/PR and PD/NR in CLL patients (*n* = 12) and (**d**) CR/PR and PD/NR in MM patients (*n* = 19). Abbreviations—CLL: chronic lymphocytic leukemia; CR: complete response; MM: multiple myeloma; NR: no response; PR: partial response; qPCR: quantitative polymerase chain reaction; T_all_: the sum of all measured CAR-T cell phenotypes. *: *p* ≤ 0.05, **: *p* ≤ 0.01.

Log_10_-transformed CCS using C_max_ values for all CAR-T cells derived from flow cytometry were reasonably correlated with log-transformed CCS using C_max_ values derived from cfDNA qPCR (*r* = 0.48, *p* = 0.037) and the CCS_qPCR_ values were significantly higher in the reference expansion population compared to the low expansion subpopulation (median: 83.7 copies µg^−1^ DNA·mL^−1^ vs. median: 4.16 copies µg^−1^ DNA·mL^−1^, *p* = 0.014) (Figure 10a,b). Furthermore, CCS_qPCR_ values were significantly higher in patients with complete response/partial response vs. patients with progressive disease/no response in previously digitized data [41] of patients with multiple myeloma [43] (*p* = 0.017) and chronic lymphocytic lymphoma [42] (*p* = 0.0051) (Figure 10c,d), further supporting our clinical composite score framework.

## 4. Discussion

Leveraging our developed population quantitative systems pharmacology CAR-T cell model, we identified patient characteristics explaining two-thirds of the large interindividual variability observed in the CAR-T cell expansion in our dataset, and these patient characteristics were also partially predictive for survival. Previously reported prognostic factors such as a high maximum CAR-T cell concentration [1,4,7,19,50], a high baseline metabolic tumor volume [50,51,52], or a high area under the concentration–time curve (AUC) in the first month [4,7,50] were not predictive for overall survival in our clinical cohort (Supplementary Figure S7–S9). Other studies similarly did not find relationships between outcome and a high maximum CAR-T cell concentration [53], a high baseline tumor burden [5,53], or a high area under the concentration–time curve (AUC) in the first month [53]. Possible reasons for the different findings could be different patient populations, different disease characteristics, different study designs, and/or different CAR-T cell products used. A pooled analysis of the clinical cohorts for which the proposed relationships were found or not found could aid in identifying potential underlying factors of the observed discrepancies. Interestingly, in a recent analysis, there was a stronger association between the probability of durable response and maximum CAR-T cells/tumor burden (*p* = 0.0017) than between the probability of durable response and maximum CAR-T cells (*p* = 0.0159) [50]. This supports our exploratory finding of the maximum CAR-T cell expansion normalized to baseline tumor burden being a more reliable predictor for outcome than maximum CAR-T cell expansion alone. Our exploratory findings and our modeling framework may spark further research regarding the impact of a previous ASCT on CAR-T cell expansion and treatment outcome.

Nonlinear mixed-effects modeling, as applied in the CAR-T cell quantitative systems pharmacology model, allows simultaneously characterizing typical profiles and identifying several layers of variability around the kinetic/dynamic parameters and observations in CAR-T cell therapy. The implementation of covariates aids in explaining parts of the interindividual variability and, thus, can increase the understanding of CAR-T cell therapy. Furthermore, our CAR-T cell modeling framework is flexible and allows the analysis of pooled datasets which offers the possibility to simultaneously leverage the information generated in multiple independent clinical studies. Therefore, especially if applied to a large and diverse clinical dataset, the developed CAR-T cell model is a valuable tool for elucidating influential factors on the kinetics and dynamics of CAR-T cell therapy and treatment outcome.

We based our structural CAR-T cell model on the T cell progressive differentiation model, which describes T cell differentiation in the order of naïve T cells via T memory stem cells (T_SCM_) over central memory cells, and over effector memory cells to short-lived effector cells. Although other lineage relationship models like the linear differentiation model [54] and the bifurcative differentiation model [55,56] have been proposed, the progressive differentiation model is most supported by experimental data [20,57,58,59,60,61]. Of note, our flow cytometry panel did not include the marker CD95 and thus did not support the detection of T_SCM_ in the presence of T_N_. Thus, using an extended staining panel, future studies including cell concentration data of both T_N_ and T_SCM_ could extend our model, considering previous reports of the positive features of T_SCM_ regarding CAR-T cell expansion and persistence. Additional flow cytometry markers such as TAM-3, LAG3, PD-1, and CD57 could further elucidate the state of the T cell with respect to exhaustion and senescence.

In previous immunotherapy models [29,62,63], T cell expansion upon tumor contact was modeled using a Michaelis–Menten equation with the tumor cell concentration in the denominator. In contrast to this, our data were described best when we used the respective T cell concentration in the denominator. Thus, our model describes that at sufficiently high T cell concentrations, T cell expansion is proportional to the tumor burden, which is in line with another previously described immunotherapy model [28]. Furthermore, this form of the equation correctly described the rapid initial CAR-T cell expansion phase followed by the sharp drop in our observed CAR-T cell concentrations. When using metabolic tumor volume in the denominator, the concentration–time profile in the expansion phase showed a lag-time and a partly mono-exponential decline after the maximum expansion. A possible reason for this could be the low initial imputed CAR-T cell doses of 0.1 cells/μL, which result in slow expansion if the expansion term postulates that expansion is proportional to the concentration of CAR-T cells at high tumor burdens. Plots for the predicted concentration–time profiles for the base models (i.e., not considering covariates or mixture model) using either metabolic tumor volume or the respective CAR-T cell population in the denominator are shown in Supplementary Figure S10. The expansion term should be revisited once a larger sample size is available to ensure that expansion can be well-described over a broad range of initial tumor burdens and that no unphysiological expansion rates are postulated. Furthermore, it is possible that using the tumor burden in the denominator as proposed by several other researchers [29,62,63] would describe the trajectory equally well or even better than our suggested term when using individual CAR-T cell concentrations in the pre-distribution phase, and this should be explored upon availability of such data.

Not in our study, but if lymphodepleting chemotherapy was administered in higher doses and for longer than three days, it may exert some decreasing effect on the metabolic tumor volume. A prerequisite for the identification of separate, rapid killing effect parameters to comprehensively characterize this process is data from multiple tumor volume assessments, i.e., prior to and after lymphodepleting chemotherapy.

Based on T cell physiology, we aimed to determine different values for the baseline maximum expansion rate upon tumor contact V_max,base_ for each phenotype by estimating V_max,base_ for T_N_ and fractional changes in V_max,base_ for the remaining three phenotypes. Point estimates of the fractional changes in V_max,base_ for each phenotype were plausible (T_CM_: +24%; T_EM_: +14%, T_Eff_: −79%), however, with relative standard errors of 130–583%, the estimates were imprecise. We thus simplified the model by assuming the same V_max,base_ for T_N_, T_CM_, and T_EM_ and removing the respective expansion term for T_Eff_. Additional in vivo and in vitro data need to become available for precise estimation of V_max,base_ parameter values for each CAR-T cell phenotype and for identification of the best CAR-T cell phenotype(s) for the strongest expansion.

We identified two covariates that significantly influenced the baseline maximum expansion rate V_max1,base_, namely the CD4^+^/CD8^+^ CAR-T cell ratio at day seven and a previous ASCT. By additionally considering if patients showed a reference or low baseline expansion, we could substantially reduce the estimated interindividual variability on V_max1,base,ref_ by two-thirds from 446% to 150% CV. We identified V_max1,base,ref_ to moderately decrease with a higher ratio of CD4^+^ to CD8^+^ T cells at day seven. This means that we estimated CD8^+^ T cells to have a higher expansion rate than CD4^+^ T cells, as reported previously [64]. In contrast, we did not identify covariates that could explain parts of the large interindividual variability on CAR-T cells’ maximum tumor killing rate (V_max5_). While we did find a significant positive relationship between cytokine release syndrome grade ≥2 and maximum tumor killing rate by T_CM_ (V_max5,2_), we think that this relationship is rather due to correlation than causation. A higher immune activation, leading to a higher grade of cytokine release syndrome, might be the reason for the increased killing rate. However, as we aimed for our model to be mechanistic and our data did not include the link (i.e., a biomarker) between cytokine release syndrome and V_max5,2_, we decided not to include cytokine release syndrome as a covariate on V_max5_. In general, the units of model parameters V_max1_, V_max5,2_, and the CCS could be further transformed by resolving the units, e.g., the different volume units (µL) and (mL). However, to ease interpretability and retain awareness for the different origins of the units ((µL) represents the distribution volume of the CAR-T cells and (mL) represents the metabolic tumor volume), the units of the parameters were kept in their original form.

Even though our model described the observed clinical data very well and allowed elucidating sources of interindividual variability, rather high residual unexplained variability of 59–120% CV remained. Data of additional patients and a higher number of samples per patients will allow optimizing parameter estimates and further investigating potential covariates to reduce the residual unexplained variability.

Leveraging our model, we identified a subpopulation with low maximum T cell expansion upon tumor contact. This subpopulation consisted of 20% patients (*n* = 4 of 19) in our dataset, which is comparable to a previous clinical study in which 12% (*n* = 5 of 43 patients) of patients showed low T cell expansion and had a poor prognosis [10]. T cells of these patients showed an increased frequency of LAG3^+^/TNFα_lo_ T cells in the manufacturing product and rapid expression of exhaustion markers after infusion. We hypothesize that the same pattern could have been observed in patients of our low expansion subpopulation. Unfortunately, no exhaustion markers were measured in our dataset, so we were not able to investigate this further, but the generated hypothesis should be tested in future. Furthermore, while we observed strong trends for differences in survival between the model-defined reference expansion and the low expansion (sub)population (PFS: 11 months vs. 2.5 months, OS: not reached vs. four months), the differences were not significant. Among the low expansion subpopulation, there was one individual with a previous ASCT, very high baseline metabolic tumor volume, and ~40-fold higher CAR-T cell C_max_ compared to the mean C_max_ in the other 18 patients. Interestingly, this individual’s survival was also much longer than the rest of the low expansion subpopulation (ongoing response at 16 months vs. median PFS and OS of 2 months). Had this patient been excluded from the analysis, the differences in PFS and OS between both (sub)populations would have been highly significant (*p* = 0.021 and 0.0049, respectively). Thus, future studies with a larger sample size to investigate the cell kinetic-independent impact of ASCT and other factors on survival and the model’s potential for response prediction are highly warranted. We subsequently translated our predictive model parameter V_max1_ into clinical composite scores (CCS) of maximum CAR-T cell concentrations/baseline metabolic tumor burden, measurable in the clinic. The excellent correlations between V_max1_ and the CCS for all CAR-T cell subpopulations (r ≥ 0.86) support our model. The highest concordance between the CCS for T_N_ (r = 0.98) was supported by a previous correlative analysis [50] and allowed us to determine a CCS_TN_ cut-off value for early response prediction.

We are aware of our small sample size, resulting from CAR-T cell therapy being a very new (and expensive) therapy in its infancy of a per se very small patient population; thus, only limited data are available worldwide overall. Furthermore, no exhaustion markers were measured. Yet, the exploratory findings of our QSP model allowed the generation of various hypotheses which should be tested with further data to arise in future. In addition, the model might support the design of those experiments/studies well, i.e., which data to sample at which timepoints, to gain maximal information from the data. Importantly, we achieved precise estimation of model parameters in our cohort and our clinical composite score framework was supported by a recent correlative analysis [50]. Nevertheless, future studies with a larger sample size and measuring exhaustion markers will have to be performed to test our hypotheses, revise, and refine our model parameters and determine a robust CCS cut-off value. The improved outcome observed in our dataset in patients with ASCT also needs to be investigated further in a larger study focusing on whether prior ASCT increases T cell expansion and survival. Possible reasons are beneficial disease characteristics in patients eligible for ASCT, the removal of an immunosuppressive microenvironment [12], and the availability of ‘fitter’ T cells that have not been damaged by previous cycles of chemotherapy. Concerning the latter point and since not every patient can undergo ASCT, a possible strategy could be to collect T cells before the start of chemotherapy and, depending on treatment response, utilize them for CAR-T cell manufacturing as needed. Furthermore, we hypothesize that a minimal time difference between the last cycle of chemotherapy and T cell collection has to be maintained, allowing T cells to recover and increasing the chance for high T cell fitness in the manufacturing product. Thus, future studies will focus on determining the optimal time differences between the last cycle of chemotherapy and T cell collection and between ASCT and T cell collection. With more data on these aspects to be collected, our QSP model can be expanded to consider the fitness of T cells prior to manufacturing and thus the potential for a robust expansion upon infusion.

Finally, it would be advantageous to predict outcomes before CAR-T cell infusion, or even before CAR-T cell manufacturing. Thus, future studies applying our QSP model on data including (i) patient baseline characteristics, (ii) CAR-T cell fitness in the leukapheresis product, (iii) CAR-T cell fitness in the infusion product, (iv) metabolic tumor volume over time, (v) CAR-T cell concentrations in vivo over time, and (vi) clinical endpoints could identify and quantify further predictors for long-term response and in the best-case scenario allow response prediction before CAR-T cell manufacturing.

## 5. Conclusions

In conclusion, our innovative quantitative systems pharmacology CAR-T cell model allowed us to describe the concentration–time profiles of different CAR-T cell phenotypes and metabolic tumor volume in a clinical dataset in a coherent framework. Using the model, we identified factors explaining two-thirds of the high interindividual variability in CAR-T cell expansion and survival in our clinical dataset. Once validated in a larger clinical cohort, the developed quantitative systems pharmacology CAR-T cell model can be used to further identify yet unknown factors resulting in the highly variable kinetics and dynamics of CAR-T cell therapy.

## Figures and Tables

**Table 1:** Final parameter estimates for the CD19-specific CAR-T cell quantitative systems pharmacology model.

Parameter (Unit)	Description	Estimate	RSE or Literature Source
V_max1,base,ref_ [(cells·µL^−1^) ·day^−1^·mL^−1^]	Maximum expansion rate per mL tumor volume of T_N_, T_CM_, and T_EM_ for the reference expansion population without previous ASCT and a CD4^+^/CD8^+^ CAR-T cell ratio at day seven of 1	0.00846	36%
V_max1,base,low_ [(cells·µL^−1^) ·day^−1^·mL^−1^]	Maximum expansion rate per mL tumor volume of T_N_, T_CM_, and T_EM_ for the low expansion subpopulation without previous ASCT	0.000700	17%
ASCT_Vmax1_ § (−)	Fractional change in V_max1,base,ref_ or V_max1,base,low_ due to a previous ASCT	2.53	31%
CD4/CD8_exp_ † (−)	Fractional change in V_max1,base,ref_ due to a change of the CD4^+^/CD8^+^ CAR-T cell ratio on day seven from a value of 1	−0.385	45%
KM_1_ (cells·µL^−1^)	T_N_, T_CM_, or T_EM_ concentration at half-maximum expansion of T_N_, T_CM_, or T_EM_	1.13	22%
kp_1_ (day^−1^)	Homeostatic proliferation rate constant for T_N_	0.0005	[47]
kp_2_ (day^−1^)	Homeostatic proliferation rate constant for T_CM_	0.007	[47]
kp_3_ (day^−1^)	Homeostatic proliferation rate constant for T_EM_	0.007	[47]
k_12_ (day^−1^)	Rate constant for differentiation of T_N_ to T_CM_	0.140	9%
k_23_ (day^−1^)	Rate constant for differentiation of T_CM_ to T_EM_	0.191	11%
k_34_ (day^−1^)	Rate constant for differentiation of T_EM_ to T_Eff_	0.355	13%
ke_1_ (day^−1^)	Death rate constant for T_N_	0.0104 ‡	13%
ke_2_ (day^−1^)	Death rate constant for T_CM_	0.0104 ‡	13%
ke_3_ (day^−1^)	Death rate constant for T_EM_	0.0104 ‡	13%
ke_4_ (day^−1^)	Death rate constant for T_Eff_	0.518	13%
V_max 5,1_ [mL·day^−1^ ·(cells·µL^−1^)^−1^]	Maximum killing rate of metabolic tumor volume by T_N_	2.57 *	39%
V_max 5,2_ [mL·day^−1^ ·(cells·µL^−1^)^−1^]	Maximum killing rate of metabolic tumor volume by T_CM_	4.04	39%
V_max 5,3_ [mL·day^−1^ ·(cells·µL^−1^)^−1^]	Maximum killing rate of metabolic tumor volume by T_EM_	3.78 *	39%
V_max 5,4_ [mL· day^−1^ ·(cells·µL^−1^)^−1^]	Maximum killing rate of metabolic tumor volume by T_Eff_	4.24 *	39%
KM_5_ (mL)	Metabolic tumor volume at half-maximum killing rate	276	33%
K_0_ (mL)	Maximum tumor volume observable (tumor carrying capacity)	5000	-
k_5_ (day^−1^)	Proliferation rate constant of metabolic tumor volume	0.0023	-
MIXP (−)	Estimated proportion of patients in the reference population using the mixture model	0.803	11%
IIV V_max1,base,ref_	Interindividual variability in V_max1,base,,ref_	150% CV	19%
IIV V_max 5,2_	Interindividual variability in V_max 5,2_	307% CV	19%
RUV T_N_	Residual unexplained variability in observed T_N_ concentrations	59.1% CV	11%
RUV T_CM_	Residual unexplained variability in observed T_CM_ concentrations	85.9% CV	9%
RUV T_EM_	Residual unexplained variability in observed T_EM_ concentrations	120% CV	9%
RUV T_Eff_	Residual unexplained variability in observed T_Eff_ concentrations	70.6%CV	10%
RUV CD19^+^ tumor	Residual unexplained variability in observed metabolic tumor volumes	115% CV	12%

IIV: interindividual variability; *RSE*: relative standard error, % = (standard error/estimate)·100; §: implemented as fractional change covariate model, †: implemented as power covariate model; ‡ derived using the estimated death rate constant for effector T cells ke_4_ and the relationship between death rate constants of short- and long-lived T cells in the publication by Stein et al. [21]; * derived using the estimated maximum killing rate of metabolic tumor volume by T_CM_ and the digitized relationships of tumor cell killing rates in the publication by Schmueck-Henneresse et al. [49].

## Data Availability

The datasets generated during and/or analyzed during the current study are not publicly available because patients did not provide consent for sharing their data in a public database and this purpose was also not included in the IRB proposal. On reasonable request, the datasets are available from corresponding author SSN. The NONMEM model code for the population quantitative systems pharmacology CAR-T cell model are available on reasonable request from corresponding author CK.

## Supplementary Materials

The following are available online at https://www.mdpi.com/article/10.3390/cancers13112782/s1. Figure S1: Flow cytometry gating strategy for the isolation of CD4^+^ and CD8^+^ CAR-T cells from peripheral blood samples of patients at days 7, 14, and 28 after infusion and identification of the different phenotypes. Figure S2: Digitized data on C_max_ and Baseline tumor burden in patients with MM and CLL. Figure S3: Measured concentrations and simulated typical and individual model predictions of different species after CAR-T-cell infusion for patients in the low expansion subpopulation before and after implementation of the mixture model. Figure S4: Estimated maximum expansion capacity upon tumor contact parameter Vmax_1_ versus the CD4/CD8 CAR-T cell ratio at day seven. Figure S5: Simulated typical CAR-T cell concentration–time profiles using different initial CAR-T cell concentrations (0.1 cells µL^−1^ as used in our model (light blue) and ten-fold lower (dark blue) or ten-fold higher (purple) and a baseline metabolic tumor volume of 85.7 mL (median baseline metabolic tumor volume in our dataset) (assuming reference covariate values of no previous autologous stem cell transplantation and a CD4/CD8 CAR-T cell ratio of 1).Figure S6: Receiver operating characteristic (ROC) curve for deriving an optimal cut-off value of the clinical composite score (CCS) Maximum CAR-T_N_ cell concentrations (C_max_)/Baseline metabolic tumor volume [(cells · µL^−1^) · mL^−1^] to determine if patients belong to low expansion subpopulation. Figure S7: Kaplan–Meier plots for and progression-free survival and overall survival in patients with high and low metabolic tumor volume in mL at baseline. Figure S8: Kaplan–Meier plots for progression-free survival and overall survival in patients with high (above median) and low (lower or equal to median) maximum CAR-T cell concentrations. Data points: measured concentrations. Figure S9: Kaplan–Meier plots for progression-free survival and overall survival in patients with high (above median) and low (lower or equal to median) AUC_0–28d_ CAR-T cell concentrations. Figure S10: Measured CAR-T cell concentrations and predicted concentration–time profiles using base models with different forms of CAR-T cell expansion terms. Table S1: Patient characteristics. Table S2: Observed and predicted CAR-T cell kinetic parameters in the reference population and the low expansion subpopulation.
