# Supplementary material for: Early Survival Prediction Framework in CD19-Specific CAR-T Cell Immunotherapy Using a Quantitative Systems Pharmacology Model
Source: Cancers (Basel). 2021 Jun 3;13(11):2782. doi: 10.3390/cancers13112782 (PMC8199881; doi:10.3390/cancers13112782)
Supplement: Supplementary file 1 [file cancers-13-02782-s001.zip › cancers-1181067-supplementary.pdf]

cancers

MDPI

Supplementary Metrials: Early response prediction framework in CD19-specific CAR-T cell immunotherapy using a quantitative systems pharmacology model

Anna Mueller-Schoell, Nahum Puebla-Osorio, Robin Michelet, Michael Green, Annette Künkele, Wilhelm Huisinga, Paolo Strati, Beth Chasen, Sattva S. Neelapu, Cassian Yee and Charlotte Kloft

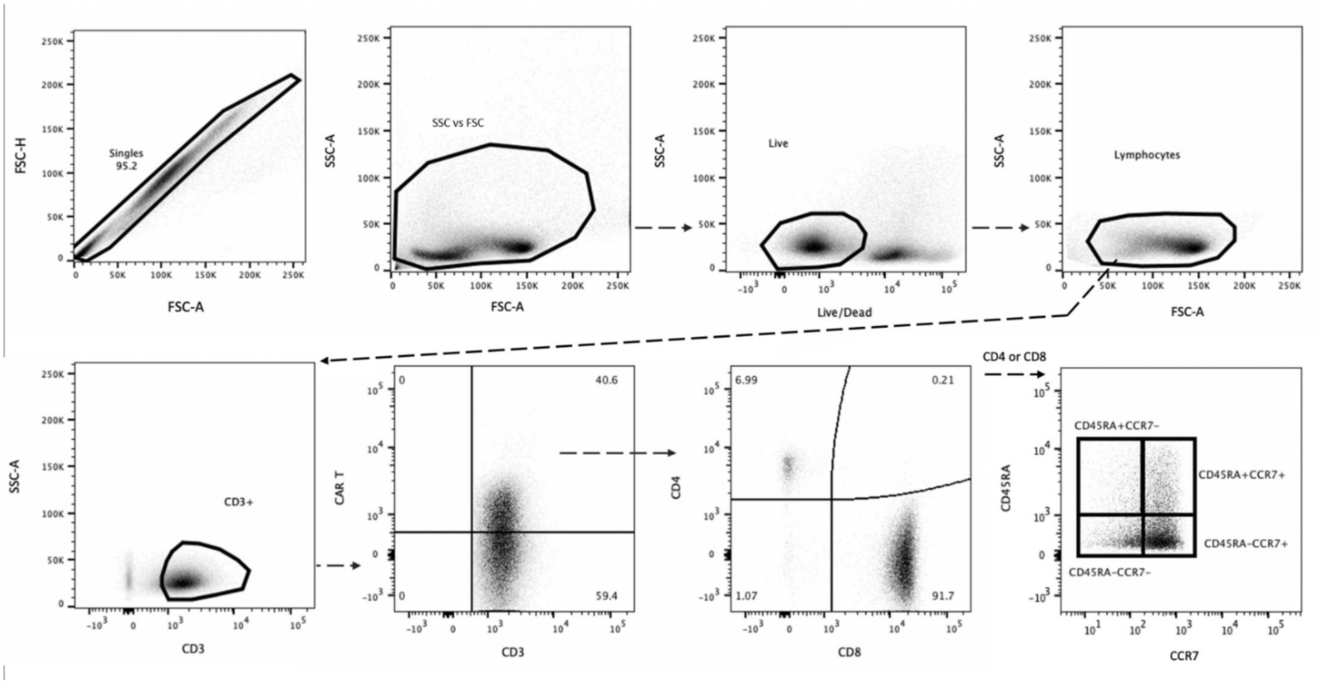

Figure S1. Flow cytometry gating strategy for the isolation of CD4⁺ and CD8⁺ CAR-T cells from peripheral blood samples of patients at days 7, 14 and 28 after infusion and identification of the different phenotypes. *Abbreviations:* SSC: sideward scatter; FSC: forward scatter; CAR T: chimeric antigen receptor T cells.

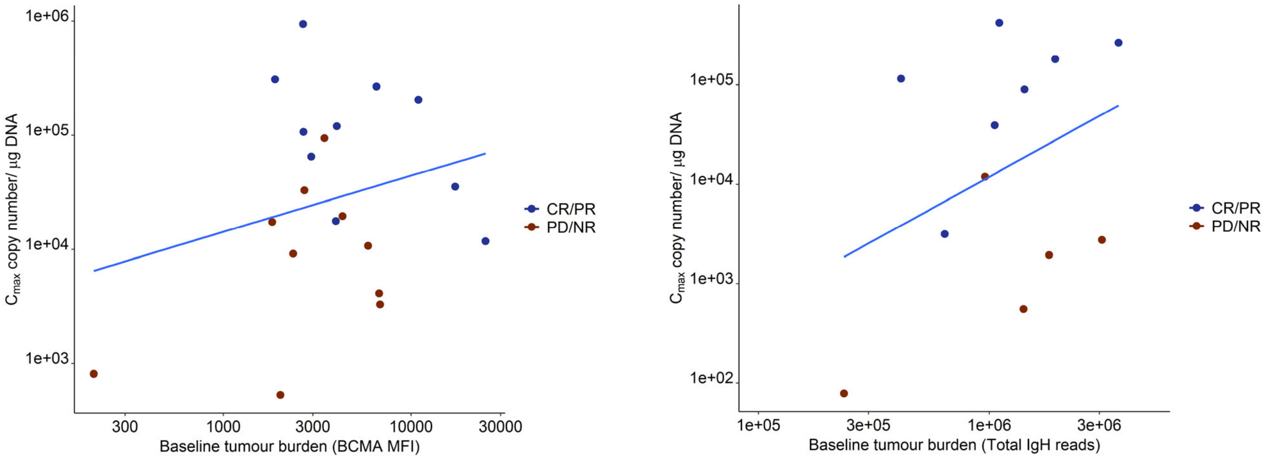

Figure S2. Digitised data on C_{max} and Baseline tumour burden in patients with MM and CLL. **Right:** MM data; **Left**: CLL data; both digitised from the publication by Liu et al. *Abbreviations:* CLL: chronic lymphocytic leukaemia; CR: complete response; MM: multiple myeloma; NR: no response; PR: partial response.

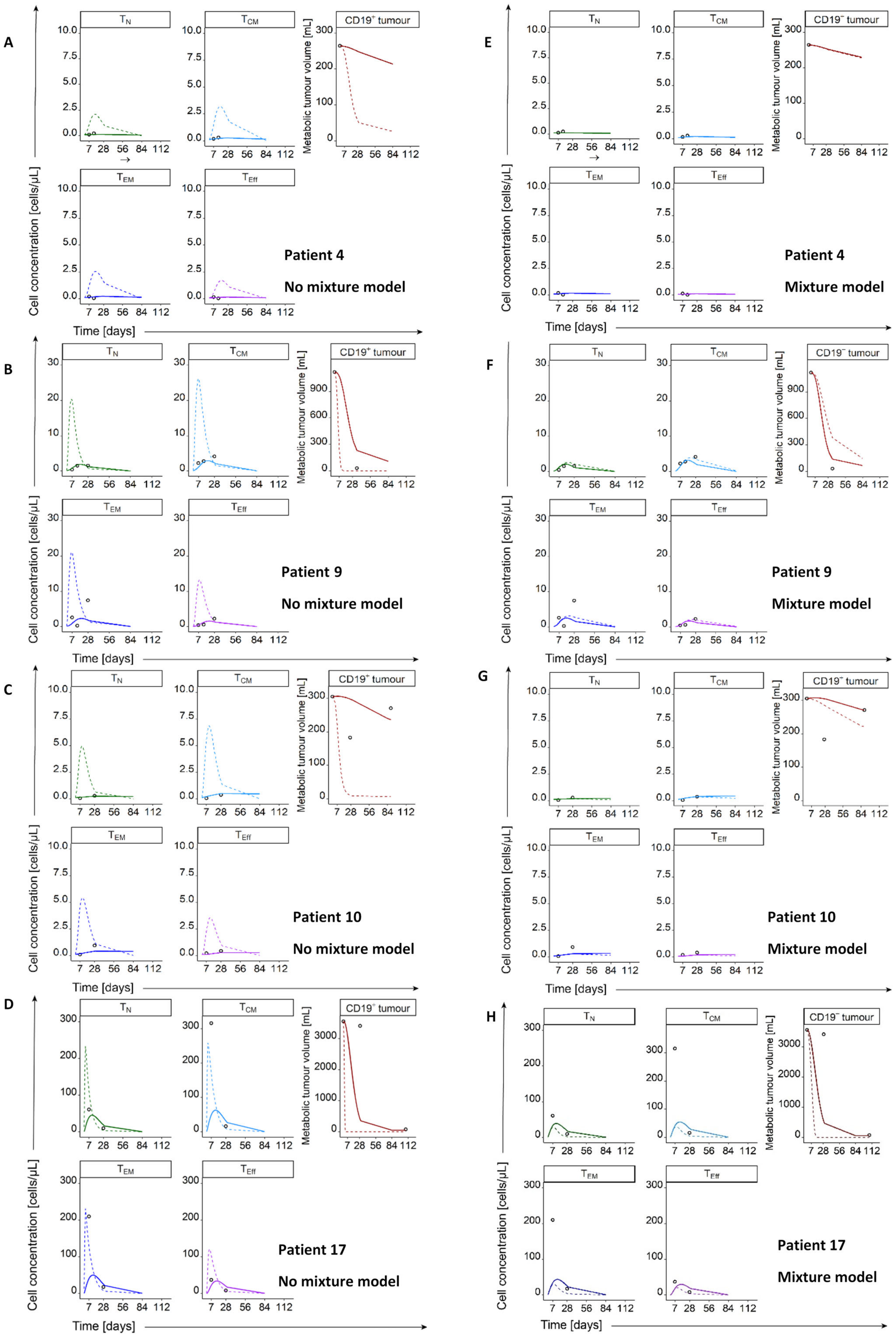
A
B
C
D
E
F
G
H
T_N
T_CM
T_EM
T_Eff
CD19⁺ tumour
CD19⁻ tumour
Cell concentration [cells/μL]
Metabolic tumour volume [mL]
Time [days]
Patient 4
No mixture model
Mixture model
Patient 9
No mixture model
Mixture model
Patient 10
No mixture model
Mixture model
Patient 17
No mixture model
Mixture model

Figure S3. Measured concentrations and simulated typical and individual model predictions of different species after CART-cell infusion for patients in the low expansion subpopulation before and after implementation of the mixture model. *Data points*: measured concentrations. *Dashed lines*: simulated typical model predictions. *Solid lines*: individual model predictions. Panels (**A**)-(**D**) show individual plots using the original model and panels (**E**)-(**H**) show individual plots using the mixture model. Abbreviations: T_N: Naïve T cells, T_{CM}: central memory T cells, T_{EM}: effector memory T cells, T_{Eff}: Effector T cells, $CD19^+$ tumour: $CD19^+$ metabolic tumour volume.

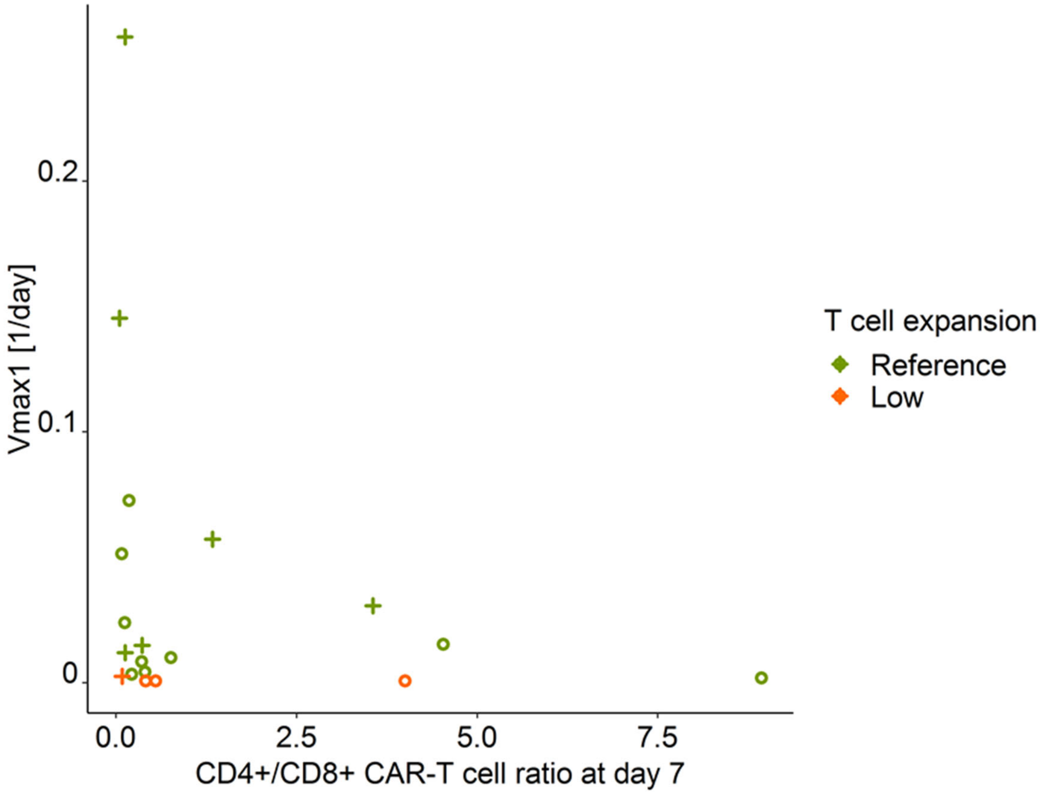

Figure S4. Estimated maximum expansion capacity upon tumor contact parameter $Vmax_1$ versus the CD4/CD8 CAR-T cell ratio at day seven. Circles: no previous autologous stem cell transplantation, plus signs: previous autologous stem cell transplantation.

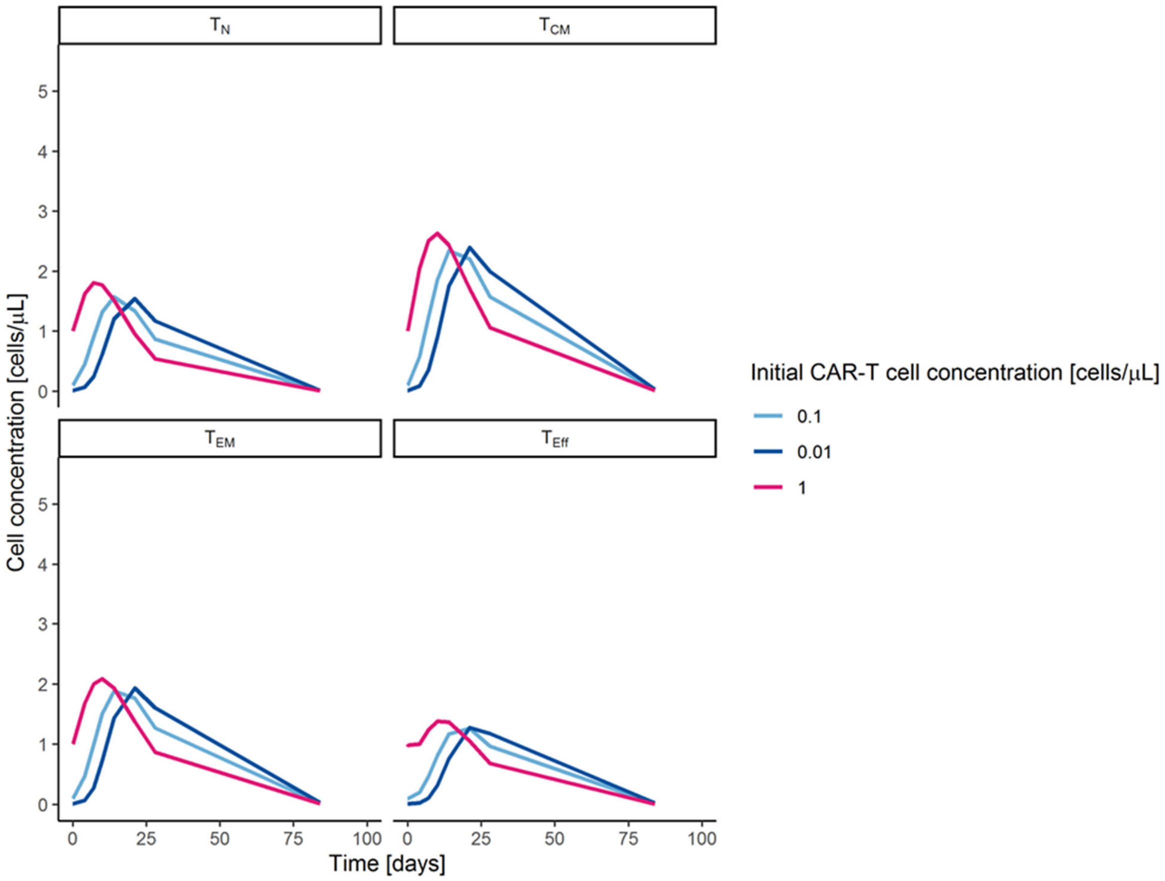

Figure S5. Simulated typical CAR-T cell concentration-time profiles using different initial CAR-T cell concentrations (0.1 cells μL^{-1} as used in our model (light blue) and ten-fold lower (dark blue) or ten-fold higher (purple) and a baseline metabolic tumour volume of 85.7 mL (median baseline metabolic tumour volume in our dataset) (assuming reference covariate values of no previous autologous stem cell transplantation and a CD4/CD8 CAR-T cell ratio of 1).

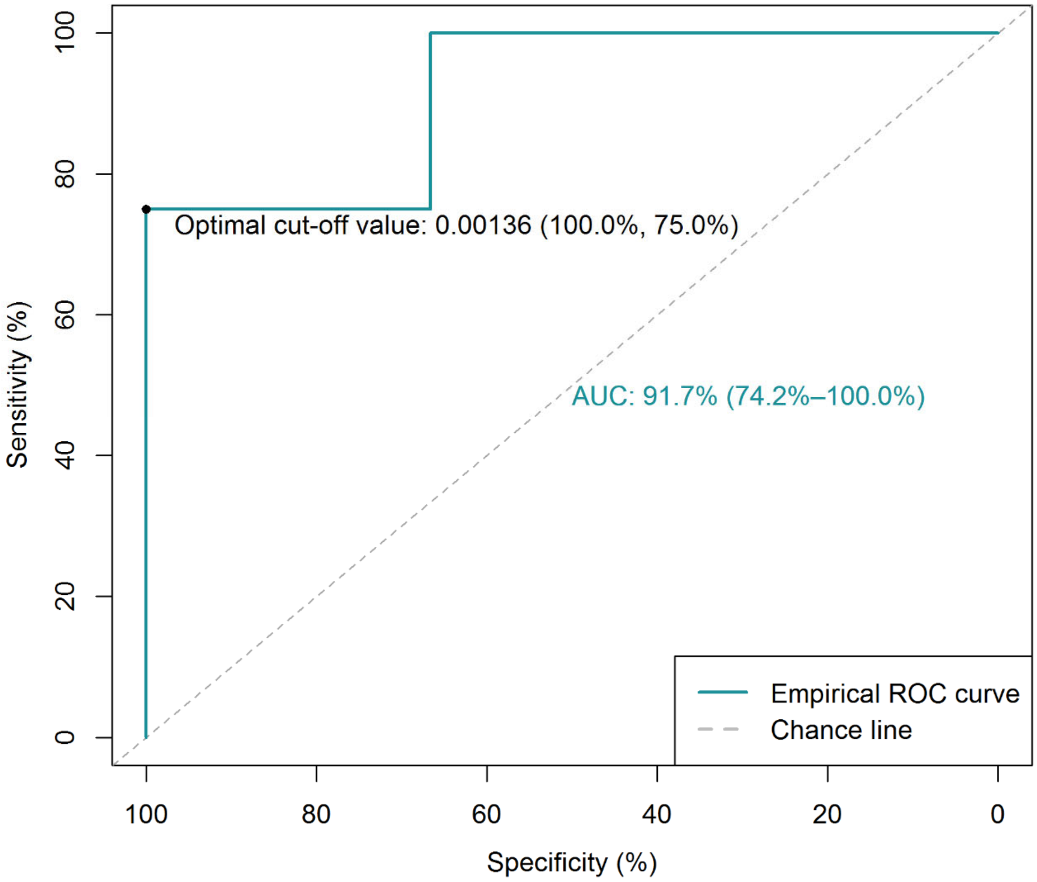

Figure S6. Receiver operating characteristic (ROC) curve for deriving an optimal cut-off value of the clinical composite score (CCS) Maximum CAR-T_N cell concentrations (C_{max})/Baseline metabolic tumour volume [(cells · μL^{-1}) · mL^{-1}] to determine if patients belong to low expansion subpopulation. The optimal cut-off value marks the value with optimal predictive capability (0.0143 [(cells · μL^{-1}) · mL^{-1}]; with 75% sensitivity, 100% specificity and an area under the curve of 91.7% (95% confidence interval: 74.2%-100%).

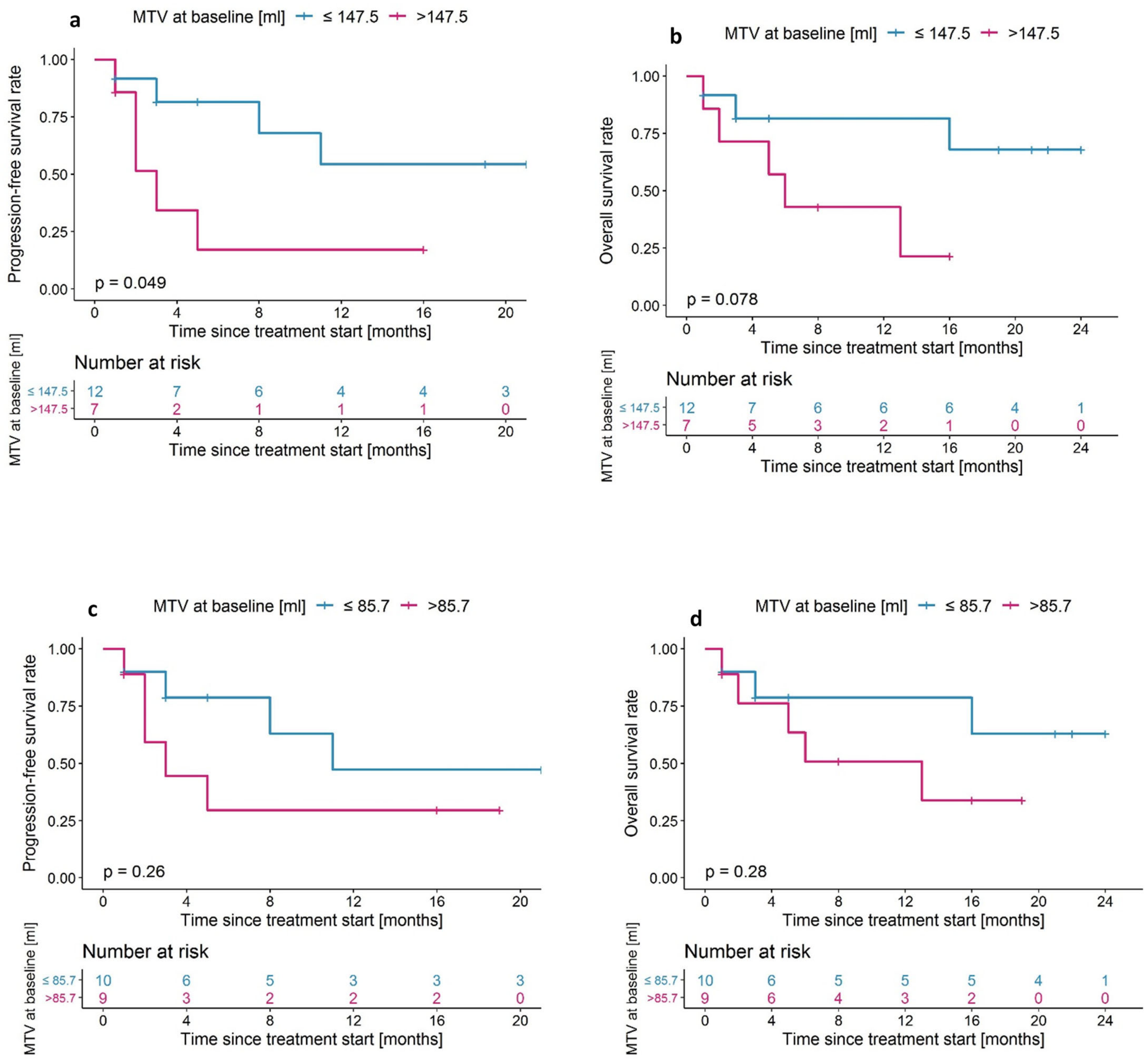

Figure S7. Kaplan-Meier plots for and progression-free survival and overall survival in patients with high and low metabolic tumor volume in mL at baseline. In plots (**a**) and (**b**), the cut-off value of 147.5 mL has been used while in plots (**c**) and (**d**), the median baseline metabolic tumour volume in our dataset has been used as cut-off value; log-rank tests. *Abbreviations:* MTV: metabolic tumour volume.

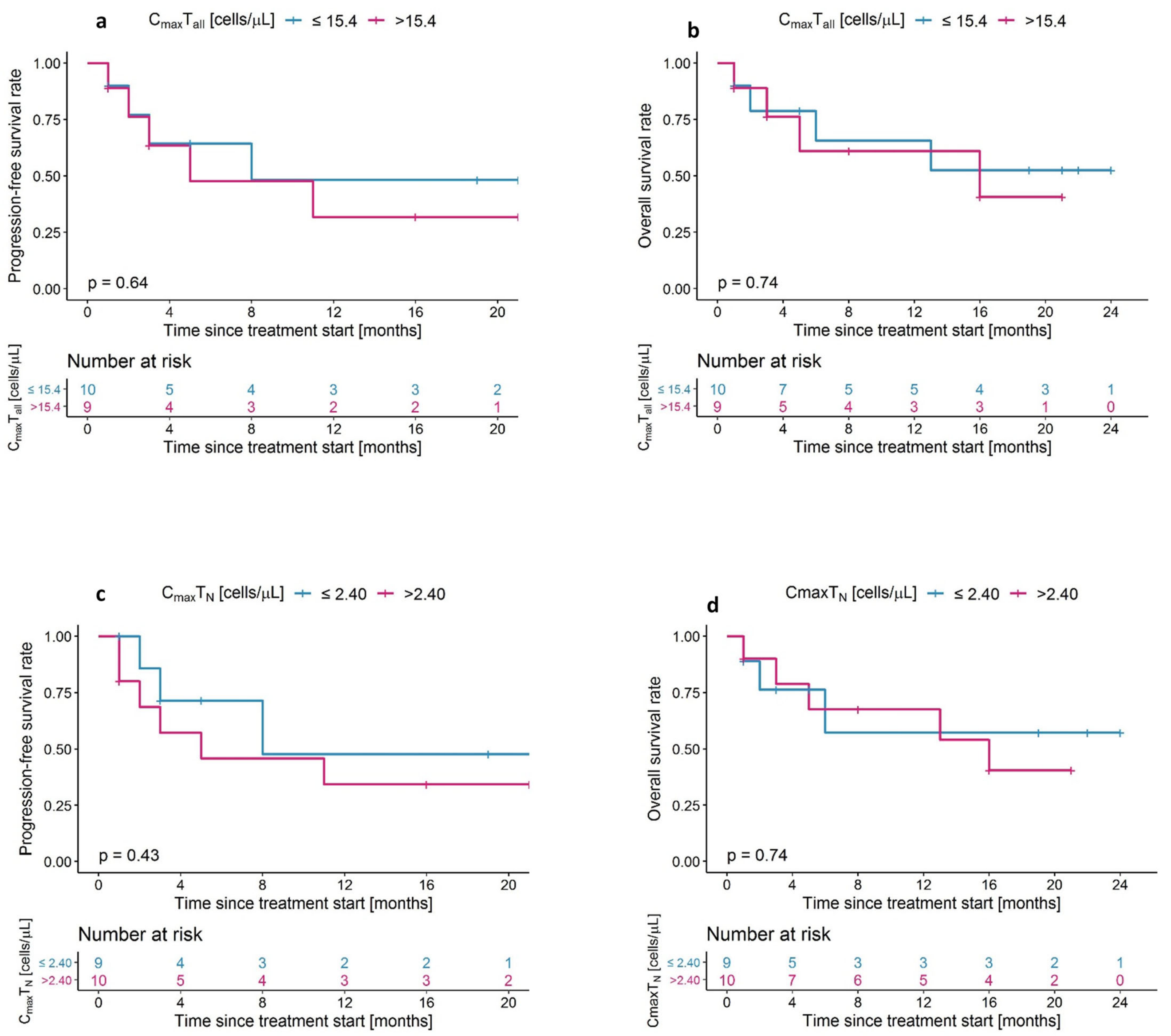

Figure S8. Kaplan-Meier plots for progression-free survival and overall survival in patients with high (above median) and low (lower or equal to median) maximum CAR-T cell concentrations. In plots (**a**) and (**n**), all CAR-T cells are assessed, while in plots (**c**) and (**d**), only naïve T cells are assessed, as the maximum concentration of naïve T cells was highest correlated with our model parameter V_{max1}; log-rank tests. *Abbreviations:* C_{max}: maximum concentration; T_{all}: the sum of all measured CAR-T cell phenotypes; T_N: naïve CAR-T cells.

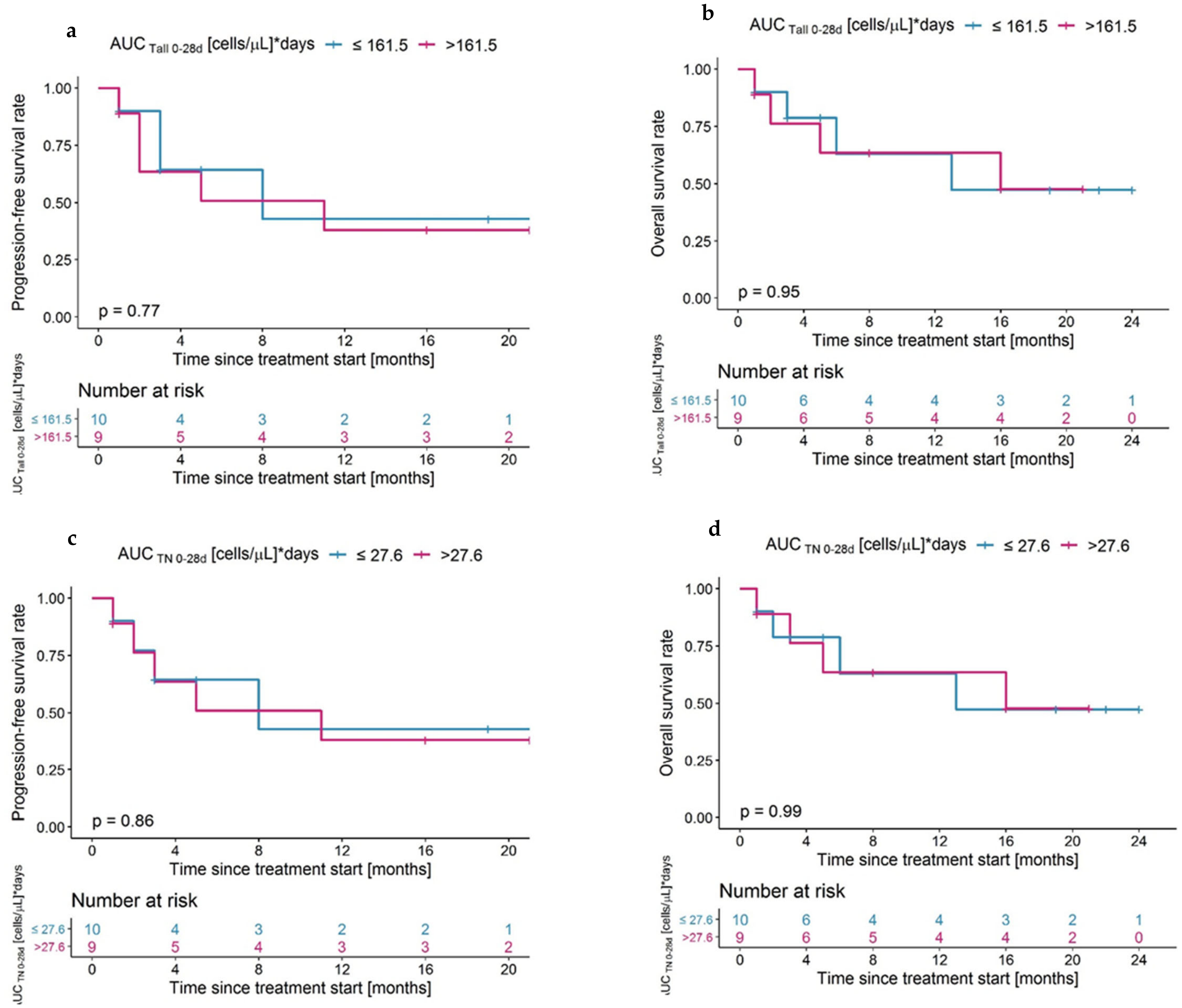

Figure S9. Kaplan-Meier plots for progression-free survival and overall survival in patients with high (above median) and low (lower or equal to median) AUC$_{0\text{-}28d}$ CAR-T cell concentrations. In plots (**a**) and (**b**), all CAR-T cells are assessed, while in plots (**c**) and (**d**), only naïve T cells are assessed, as the maximum concentration of naïve T cells was highest correlated with our model parameter V_{max1}; log-rank tests. *Abbreviations:* AUC$_{0\text{-}28d}$: area under the concentration-time curve from day 0 to day 28; T_{all}: the sum of all measured CAR-T cell phenotypes; T_N: naïve CAR-T cells.

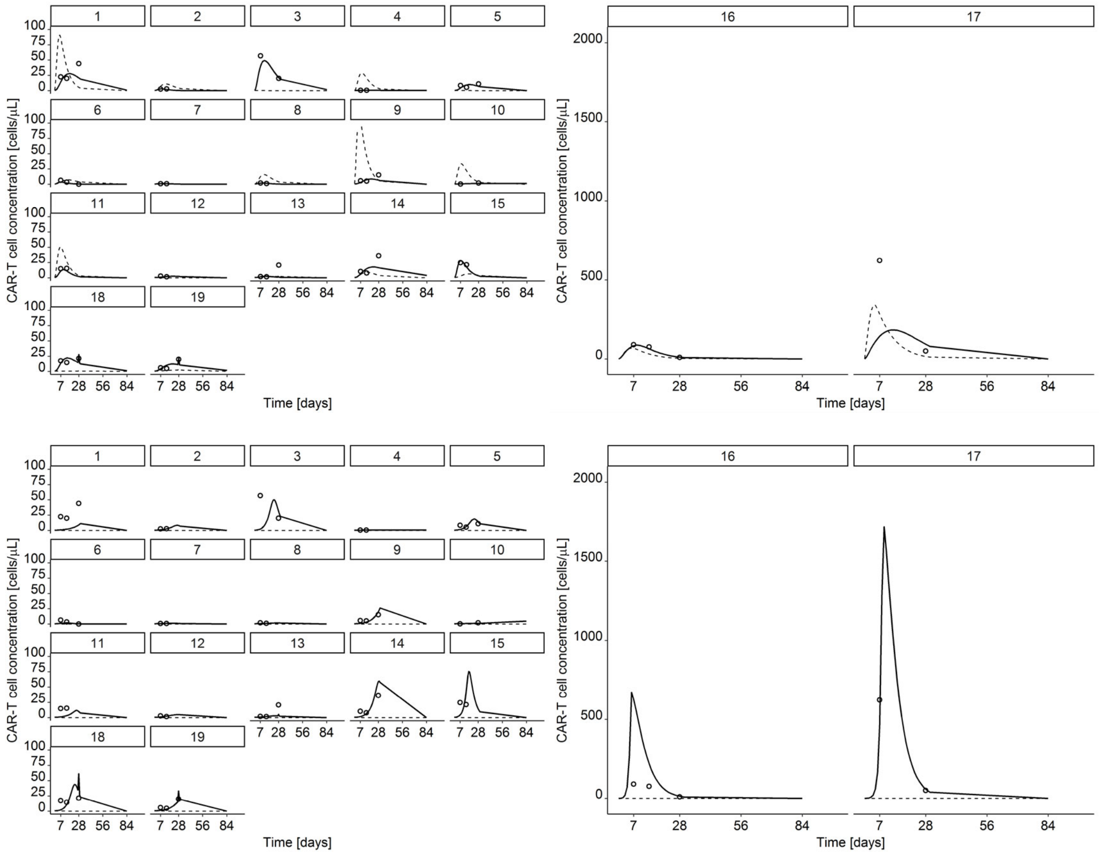

Figure S10. Measured CAR-T cell concentrations (circles) and individual (solid lines) and typical (dashed lines) model predictions using the base model (without covariates or mixture model) and using either the respective CAR-T cell population (**upper plots**) or the CD19⁺ metabolic tumour volume (**lower plots**) in the denominator of the expansion term describing CAR-T cell expansion in response to tumour contact. The base model using the respective CAR-T cell population in the denominator of the expansion term (**upper plots**) was used for the rest of the work.

Table S1. Patient characteristics.

Pat. ID	Disease Type	Sex [M/F]	Age group [years]	C_{max} naïve CAR-T cells [cells·µL^{-1}]	Baseline metabolic tumour volume [mL]	Clinical composite score $_{TN}$ * [(cells · µL^{-1}) · mL^{-1}]	Low expansion subpopulation [yes/no]	Previous ASCT [yes/no]	CD4/CD8 ratio of CAR-T cells at day seven	Progressive disease [yes/no]	PFS [months]	OS [months]	Dead [yes/no] (and reason) or lost to follow-up†
1	DLBCL	F	51-60	44.1	894	0.00542	No	No	8.94	Yes	2	8	Lost to follow-up
2	DLBCL	M	21-30	3.04	102	0.00863	No	No	0.357	No	1	1	Lost to follow-up
3	PMBCL	M	51-60	56.8	13.0	0.723	No	Yes	0.126	No	21	21	No
4	TFL	F	21-30	0.64	264	0.000870	Yes	No	4.01	No	1	1	Yes (septic shock)
5	DLBCL	F	51-60	11.0	14.2	0.169	No	Yes	1.34	No	21	21	No
6	TFL	M	31-40	6.43	70.5	0.0174	No	Yes	0.364	Yes	8	24	No
7	DLBCL	F	61-70	1.02	13.1	0.0236	No	Yes	3.56	No	22	22	No
8	PMBCL	M	71-80	2.04	142	0.00230	No	No	0.405	No	19	19	No
9	PMBCL	M	51-60	15.3	1118	0.00136	Yes	No	0.413	Yes	2	2	Yes (PD)
10	DLBCL	M	51-60	1.98	305	0.000884	Yes	No	0.549	Yes	3	6	Yes (PD)
11	DLBCL	M	61-70	15.4	479	0.00751	No	No	0.223	Yes	1	13	Yes (PD)
12	DLBCL	F	61-70	2.82	2.54	0.299	No	Yes	0.0512	No	5	5	Lost to follow-up
13	DLBCL	M	51-60	16.8	34.2	0.014	No	No	4.53	No	3	3	Lost to follow-up
14	PMBCL	M	61-70	36.2	85.7	0.0705	No	Yes	0.130	No	1	1	Lost to follow-up
15	DLBCL	M	61-70	24.7	64.1	0.164	No	No	0.0845	Yes	3	3	Yes (PD)
16	PMBCL	F	61-70	90.8	663	0.0388	No	No	0.764	Yes	5	5	Yes (PD)
17	DLBCL	M	51-60	623	3555	0.017	Yes	Yes	0.0866	No	16	16	No
18	PMBCL	M	51-60	17.3	20.0	0.224	No	No	0.182	Yes	1	1	Yes (PD)
19	DLBCL	M	51-60	15.8	32.5	0.124	No	No	0.126	Yes	11	16	Yes (PD)

Abbreviations: ASCT: autologous stem cell transplantation; C_{max}: maximum concentration; DLBCL: Diffuse large B cell lymphoma; F: female; M: male; OS: overall survival; PD: progressive disease; PFS: progression-free survival; Pat: patient; PMBCL: primary mediastinal B-cell lymphoma; TFL: transformed follicular lymphoma.

*Clinical composite score$_{TN}$: Maximum naïve CAR-T cell concentrations/Baseline metabolic tumour volume

†Patients who were lost to follow-up were from overseas or other US states and did not return to MD Anderson Cancer Center for further monitoring.

Table S2. Observed and predicted CAR-T cell kinetic parameters in the reference population and the low expansion subpopulation.

Pharmacokinetic parameter	Reference population (*n* = 15)	Low expansion subpopulation (*n* = 4)
**Observed CAR-T cell kinetic parameters in the reference population and the low-expansion subpopulation**		
Maximum CAR-T cell concentration (C_{max}) [cells·µL^{-1}]	Median: 15.8 Range: 1.02–90.8	Median: 8.64 Range: 0.64–623
Area under the concentration-time curve from day 0 to day 28 ($AUC_{0\text{-}28d}$) [(cells·µL^{-1}) · day^{-1}]	Median: 162 Range: 6.48–1195	Median: 102 Range: 4.34–7068
Time at maximum CAR-T cell concentration (T_{max}) [day]	Median: 14 Range: 7–28	Median: 17.5 Range: 7–28
C_{max}/Baseline metabolic tumour volume [(cells·µL^{-1})·mL^{-1}]	Median: 0.386 Range: 0.0143–4.38	Median: 0.0101 Range: 0.00242–0.175
**Model-predicted CAR-T cell kinetic parameters in the reference population and the low-expansion subpopulation**		
Maximum CAR-T cell concentration (C_{max}) [cells·µL^{-1}]	Median: 13.8 Range: 1.05 –79.9	Median: 5.231 Range: 0.619–166
Area under the concentration-time curve from day 0 to day 28 ($AUC_{0\text{-}28d}$) [(cells·µL^{-1}) · day^{-1}]	Median: 179 Range: 11.3–1271	Median: 94.0 Range: 6.41–2765
Time at maximum CAR-T cell concentration (T_{max}) [day]	Median: 12 Range: 7–28	Median: 23.5 Range: 14–30
C_{max}/Baseline metabolic tumour volume [(cells·µL^{-1})·mL^{-1}]	Median: 0.121 Range: 0.0136–3.59	Median: 0.00594 Range: 0.00234–0.0467
